# Development of Human Pituitary Neuroendocrine Tumor Organoids to Facilitate Effective Targeted Treatments of Cushing’s Disease

**DOI:** 10.3390/cells11213344

**Published:** 2022-10-23

**Authors:** Jayati Chakrabarti, Ritu Pandey, Jared M. Churko, Jennifer Eschbacher, Saptarshi Mallick, Yuliang Chen, Beth Hermes, Palash Mallick, Ben N. Stansfield, Kelvin W. Pond, Curtis A. Thorne, Kevin C. J. Yuen, Andrew S. Little, Yana Zavros

**Affiliations:** 1 Department of Cellular and Molecular Medicine, University of Arizona College of Medicine, Tucson, AZ 85721, USA; 2 Center for Biomedical Informatics and Biostatistics, University of Arizona Health Sciences, Tucson, AZ 85721, USA; 3 Department of Neuropathology, Barrow Neurological Institute, Phoenix, AZ 85013, USA; 4 University of Arizona Cancer Center Bioinformatics Core, Tucson, AZ 85721, USA; 5 Department of Neuroendocrinology, Barrow Neurological Institute, Phoenix, AZ 85013, USA; 6 Department of Neurosurgery, Barrow Neurological Institute, Phoenix, AZ 85013, USA

**Keywords:** organoids, neuroendocrine tumors, induced pluripotent stem cells, *CDH23*

## Abstract

(1) Background: Cushing’s disease (CD) is a serious endocrine disorder caused by an adrenocorticotropic hormone (ACTH)-secreting pituitary neuroendocrine tumor (PitNET) that stimulates the adrenal glands to overproduce cortisol. Chronic exposure to excess cortisol has detrimental effects on health, including increased stroke rates, diabetes, obesity, cognitive impairment, anxiety, depression, and death. The first-line treatment for CD is pituitary surgery. Current surgical remission rates reported in only 56% of patients depending on several criteria. The lack of specificity, poor tolerability, and low efficacy of the subsequent second-line medical therapies make CD a medical therapeutic challenge. One major limitation that hinders the development of specific medical therapies is the lack of relevant human model systems that recapitulate the cellular composition of PitNET microenvironment. (2) Methods: human pituitary tumor tissue was harvested during transsphenoidal surgery from CD patients to generate organoids (hPITOs). (3) Results: hPITOs generated from corticotroph, lactotroph, gonadotroph, and somatotroph tumors exhibited morphological diversity among the organoid lines between individual patients and amongst subtypes. The similarity in cell lineages between the organoid line and the patient’s tumor was validated by comparing the neuropathology report to the expression pattern of PitNET specific markers, using spectral flow cytometry and exome sequencing. A high-throughput drug screen demonstrated patient-specific drug responses of hPITOs amongst each tumor subtype. Generation of induced pluripotent stem cells (iPSCs) from a CD patient carrying germline mutation CDH23 exhibited dysregulated cell lineage commitment. (4) Conclusions: The human pituitary neuroendocrine tumor organoids represent a novel approach in how we model complex pathologies in CD patients, which will enable effective personalized medicine for these patients.

## 1. Introduction

Cushing’s disease (CD) is a serious endocrine disorder caused by an adrenocorticotropic hormone (ACTH)-secreting pituitary neuroendocrine tumor (PitNET) that stimulates the adrenal glands to overproduce cortisol [1,2,3,4]. The WHO renamed pituitary adenomas as PitNETs [5]. While PitNETs have been defined as benign, implying that these tumors cause a disease that is not life threatening or harmful to health, in fact chronic exposure to excess cortisol has wide-ranging and detrimental effects on health. Hypercortisolism causes increased stroke rates, diabetes, obesity, depression, anxiety, and a three-fold increase in the risk of death from cardiovascular disease and cancer [4,6,7,8].

The first-line treatment for CD is pituitary surgery, which is followed by disease recurrence in 50% of patients during the 10-year follow-up period after surgery in the hands of an experienced surgeon [9,10,11]. Studies have demonstrated that surgical failures and recurrences of CD are common, and despite multiple treatments, biochemical control is not achieved in approximately 30% of patients. This suggests that in routine clinical practice, initial and long-term disease remission is not achieved in a substantial number of CD patients [7,12]. Hence, medical therapy is often considered in the following situations: when surgery is contraindicated or fails to achieve remission, or when recurrence occurs after apparent surgical remission. While stereotactic radiosurgery treats incompletely resected or recurrent PitNETs, the main drawbacks include the longer time to remission (12–60 months) and the risk of hypopituitarism [3,13,14]. There is an inverse relationship between disease duration and reversibility of complications associated with the disease, thus emphasizing the importance of identifying an effective medical strategy to rapidly normalize cortisol production by targeting the pituitary adenoma [4,7,12]. Unfortunately, the lack of current standard of care treatments with low efficacy and tolerability makes CD a medical therapeutic challenge.

The overall goal of medical therapy for CD is to target the signaling mechanisms to lower cortisol levels in the body [15,16]. The drugs offered for treatment of CD vary in the mechanism of action, safety, tolerability, route of administration, and drug–drug interactions [15,16]. In the era of precision medicine [17], where it is imperative to identify effective therapies early, there is an urgent need to accelerate the identification of therapies targeted to the ACTH-secreting pituitary tumor which are tailored for each individual patient. The absence of preclinical models that replicate the complexity of the PitNET microenvironment has prevented us from acquiring the knowledge to advance clinical care by implementing therapies specifically targeting the tumor, which would have a higher efficacy and tolerability for CD patients. In this instance, organoids can replicate much of the complexity of an tumor. An “organoid” is defined as a three-dimensional cell structure, grown from primary cells of dissociated pituitary tumors in Matrigel matrix, which proliferate, and differentiate in three dimensions, eventually replicating key biological properties of the tissue [18]. While pituitary cell lines predominantly represent hormonal lineages, these cultures do not reproduce the primary pituitary tissue because of the tumor transformation and non-physiological 2D culture conditions [19,20,21]. Pituitary tissue-derived organoids have been generated from mouse models [22,23]. While several human and rat pituitary spheroid/aggregate/tumoroid models have been reported, these cultures consist of poorly differentiated cells with high replicative potential which can affect drug response and produce data that poorly translate to the clinic [24,25]. In this study, we developed an organoid model derived from human PitNETs that replicated much of the cellular complexity and function of the patient’s tumor. Organoids derived from corticotroph PitNETs retained the genetic alterations of the patient’s primary tissue.

## 2. Materials and Methods

### 2.1. Generation and Culture of Human Pituitary Neuroendocrine Tumor (PitNET) Organoids

Patients with planned transsphenoidal surgery for pituitary tumors were identified in the outpatient neurosurgery clinics. Tissues were collected under the St. Joseph’s Hospital and Barrow Neurological Institute Biobank collection protocol PHXA-05TS038 and collection of outcomes data protocol PHXA-0004-72-29, with the approval of the Institutional Review Board (IRB) and patient consent. Samples were de-identified and shipped to the Zavros laboratory (University of Arizona) for processing.

Pituitary tumor tissue was collected in Serum-Free Defined Medium (SFDM) supplemented with ROCK inhibitor (Y27632, 10 µM), L-glutamine (2 mM), A83-01 (activin receptor-like kinase (Alk) 4/5/7 inhibitor, 0.5 mM), penicillin/streptavidin (1%), kanamycin (1%), amphotericin/gentamycin (0.2%), CHIR-98014 (4 mM), and thiazovivin (TZV, 2.5 mM). Tissues that contained red blood cells were incubated with Red Blood Cell (RBC) Lysis Buffer according to the manufacturer’s protocol (Thermo Fisher Scientific, San Fransisco, CA, USA). Tissues were dissected into small pieces, transferred to digestion buffer (DMEM/F12 supplemented with 0.4% collagenase 2, 0.1% hyaluronic acid, 0.03% trypsin-EDTA) and incubated for 5–10 min at 37 °C with gentle shaking. Tissue was further incubated with Accutase™ (Thermo Fisher Scientific) for 5 min at 37 °C. Enzymatically dissociated cells were pelleted and washed in DPBS supplemented with antibiotics at a 400 relative centrifugal force (RCF) for 5 min. Dissociated adenoma cells were resuspended in Matrigel™, and Matrigel™ domes containing the cells were then plated in culture dishes and overlaid with pituitary growth media (Supplemental Table S1). The culture was maintained at 37 °C at a relative humidity of 95% and 5% CO_2_. Organoid growth medium was replenished every 3–4 days and passaged after 15 days in culture.

### 2.2. Generation of Induced Pluripotent Stem Cells (iPSCs)

Induced pluripotent stem cell lines (iPSC lines) were generated from control individuals (no reported disease) or CD patients according to published protocols by the University of Arizona iPSC Core [26]. All human iPSC lines were tested and found to be negative for mycoplasma contamination using the Mycoalert Mycoplasma testing kits (LT07-318, Lonza), and no karyotype abnormalities were found (KaryoStat+, Thermo).

### 2.3. Pituitary Organoids Generated from iPSCs

Six well culture plates were coated with 2 mL/well 0.67% Matrigel (diluted in E8 media, UA iPSC core, 151169-01) and incubated at 37 °C at a relative humidity of 95% and 5% CO_2_ overnight. The iPSC lines were reprogrammed from the blood of either a healthy donor (JCAZ001) or a CD patient (iPSC^7^ and iPSC^1063^) at the University of Arizona iPSC Core. Passage 12 iPSCs were plated onto the coated plates and incubated at 37 °C at a relative humidity of 95% and 5% CO_2_. At 70% confluency, cells were passaged to freshly coated 24 well plates at a ratio of 1:8 and grown to 85–90% confluency before beginning the directed differentiation schedule. From days 0 to 3, cells were cultured in E6 media supplemented with 1% penicillin/streptomycin, 10 μM SB431542, and 5 ng/mL BMP4. BMP4 was withdrawn from the culture at day 3. Starting on day 4, the cells were cultured in E6 media, supplemented with 10 μM SB431542, 30 ng/mL human recombinant SHH, 100 ng/mL FGF8b, 10 ng/mL FGF18, and 50 ng/mL FGF10. Fifteen days after culture, the cells were harvested in cold E6 media by pipetting and resuspended in Matrigel™ (20,000 cells/50 mL Matrigel™). Matrigel™ domes containing the cells were plated in culture dishes and overlaid with differentiation media containing E6 media which was supplemented with 10 μM Y-27632, 30 ng/mL human recombinant SHH, 100 ng/mL FGF8b, 10 ng/mL FGF18, and 50 ng/mL FGF10 (Supplemental Table S2). Organoids were cultured for a further 15 days at 37 °C at a relative humidity of 95% and 5% CO_2_.

### 2.4. Spectral Flow Cytometry (Cytek™ Aurora)

The multicolor flow cytometry panel was designed using the Cytek^®^ Full Spectrum Viewer online tool to calculate the similarity index (Supplemental Figure S1). The organoids were harvested in cold SFDM media and centrifuged at 400× *g* for 5 min. Supernatant was discarded and organoids were dissociated to single cells using Accutase^®^ (Thermo Fisher Scientific 00-4555-56). The enzymatic reaction was stopped using prewarmed DPBS, and cells were then centrifuged at 400× *g* for 5 min and incubated with fluorochrome-conjugated/unconjugated primary surface or cytoplasmic antibodies (Supplemental Figure S1) at 4 °C for 30 min. Cells were then washed with Cell Staining Buffer (BioLegend # 420-201) and incubated with secondary antibodies (Supplemental Figure S1) at 4 °C for 30 min. Cells were fixed using Cytofix/Cytoperm™ Fixation/Permeabilization Solution (BD Biosciences # 554714) at 4 °C for 20 min, followed by washing with Fixation/Permeabilization wash buffer. Cells were labeled with fluorochrome-conjugated/unconjugated intracellular primary antibodies (Supplemental Figure S1) at 4 °C for 30 min, then washed and incubated with secondary antibodies at 4 °C for 30 min. Cells were resuspended in cell staining buffer and fluorescence and measured using the Cytek Aurora 5 Laser Spectral Flow Cytometer. An unstained cell sample was fixed and used as a reference control. UltraComp eBeads™, Compensation Beads (Thermo Fisher Scientific # 01-2222-42) were stained with the individual antibodies and used as single stain controls for compensation and gating. Data were acquired using the Cytek™ Aurora and analyzed using Cytobank software (Beckman Coulter, Indianapolis, IN, USA).

### 2.5. Whole Mount Immunofluorescence

Organoids were immunostained using published protocols by our laboratory [27,28,29]. Proliferation was measured by using 5-ethynyl-2′-deoxyuridine (EdU) incorporation according to the Manufacturer’s protocol (Click-IT EdU Alexa Fluor 555 Imaging Kit, Thermo Fisher Scientific C10338). Co-staining was performed by blocking fixed organoids with 2% donkey serum (Jackson Immuno Research, # 017-000-121) diluted in 0.01% PBST for 1hr at room temperature. Organoids were then incubated overnight at 4 °C with primary antibodies, followed by secondary antibodies and Hoechst (Thermo Fisher Scientific H1399, 1:1000 in 0.01% PBST) for 1 h at room temperature. Human specific primary antibodies used included: rabbit anti-ACTH (Thermo Fisher Scientific 701293, 1:250), rabbit anti-Synaptophysin (Thermo Fisher Scientific PA5-27286, 1:100), species PIT1 (Thermo Fisher Scientific PA5-98650, 1:50), rabbit anti-LH (Thermo Fisher Scientific PA5-102674, 1:100), mouse anti-FSH (Thermo Fisher Scientific MIF2709, 1:100), mouse anti-PRL (Thermo Fisher Scientific CF500720, 1:100), Alexa Flour conjugated GH (NB500-364AF647, 1:100), and mouse anti-CAM5.2 (SIGMA 452M-95, 1:250). The secondary antibodies used included Alexa Fluor 488 Donkey Anti Rabbit IgG (H+L) (Thermo Fisher Scientific A21206, 1:100) or Alexa Fluor 647 Donkey Anti Mouse IgG (H+L) (Thermo Fisher Scientific A31571, 1:100). Organoids were visualized and images were acquired by confocal microscopy using the Nikon CrestV2 Spinning Disk (Nikon, Melville, NY, USA). Fluorescence intensity and percentage of EdU positive cells of total cells, were calculated using Nikon Elements Software (Version 5.21.05, Nikon, Melville, NY, USA).

### 2.6. Nuclear Morphometric Analysis (NMA)

Nuclear Morphometric Analysis (NMA) using treated organoids was performed based on a published protocol that measures cell viability based on the changes in nuclear morphology of the cells, using nuclear stain Hoechst or DAPI [30]. Images of organoid nuclei were analyzed using the ImageJ Nuclear Irregularity Index (NII) plugin for key parameters, which included cell area, radius ratio, area box, aspect, and roundness. Using the published spreadsheet template [30], the NII of each cell was calculated with the following formula: NII = Aspect − Area Box + Radius Ratio + Roundness. The area vs. NII of vehicle-treated cells were plotted as a scatter plot using the template, and was considered as the normal cell nuclei. The same plots were generated for each condition, and the NII and area of treated cells were compared to the normal nuclei, and classified as one of the following NMA populations: Normal (N; similar area and NII), Mitotic (S; similar area, slightly higher NII), Irregular (I; similar area, high NII), Small Regular (SR; apoptotic, low area and NII), Senescent (LR; high area, low NII), Small Irregular (SI; low area, high NII), or Large Irregular (LI; high area, high NII). Cells classified as SR exhibited early stages of apoptosis, and cells classified as either I, SI, or LI exhibited significant nuclear damage. The percentage of cells in each NII classification category were calculated and plotted as a histogram using GraphPad Prism.

### 2.7. ELISA

Concentration of secreted ACTH in conditioned media that was collected from organoid cultures was measured using the Human ACTH ELISA Kit (Novus Biologicals, NBP2-66401), according to the manufacturer’s protocol. The enzyme–substrate reaction was measured spectrophotometrically (BioTek Gen5 Micro Plate Reader Version 3.11, Santa Clara, CA, USA) at a wavelength of 450 nm, and the ACTH concentration (pg/mL) was interpolated by a standard curve with a 4-parameter logistic regression analysis, using GraphPad Prism (Version 9.2.0, San Diego, CA, USA).

### 2.8. Drug Assay

Patient adenoma-derived pituitary organoids were grown in 96-well plates and treated with 147 small molecules taken from the NCI AOD9 compound library for 72 h. (https://dtp.cancer.gov/organization/dscb/obtaining/available_plates.html (accessed on 22 August 2021)). Drugs were diluted from 10 mM DMSO stock plates into 100 M DMSO working stocks with a final concentration of 1μM. All vehicle controls were treated with 0.1% DMSO. Organoid proliferation was measured using a CellTiter 96^®^ AQueous One Solution Cell Proliferation Assay kit (MTS, Promega, G3582, Madison, WI, USA) according to the manufacturer’s instruction. Organoid death was calculated based on the absorbance readings at 490 nm, collected from the MTS assay relative to the vehicle controls. Drug screens were performed with biological replicates in the same screen. Drugs were selected based on their ability to target key signaling pathways as well as clinical relevance to the treatment. Drug sensitivity is represented by cell viability, and is significant at <0.5 suppressive effect of the drugs. The percent of cell viability relative to the vehicle control was calculated. Correlation coefficients across each organoid were calculated using the Pearson method to assess confidence in replication. The variance component was detected for each drug across all organoids. A random effect model was run with a single random factor for each drug, and estimated variance was calculated by rejecting the null hypothesis that variation was not present among samples. The drug responses were grouped by variance factor, into large (vc > 100), median (100 > vc > 50), and small (vc < 50). A heatmap was used to display the differential responses in cell viability for the drugs.

Drugs that clustered together and showed response within corticotrophs were investigated further based on their mode of action. Pathways (Kegg and Reactome) and gene ontology mapping were conducted for the genes that were being targeted by the drugs, in order to evaluate the key responses in cellular processes. A network was constructed in Cytoscape v 3.8.2 (San Diego, CA, USA) for the purpose of association between the drugs and genes.

### 2.9. Drug Dose Responses

Organoids were grown in Matrigel™ domes within 96-well round-bottom culture plates. Recombinant human SHH was removed from the pituitary organoid growth media, 24 h prior to drug treatment. Organoids were treated with either vehicle (DMSO), cabergoline (Selleckchem S5842), ketoconazole (Selleckchem S1353), roscovitine (Selleckchem S1153), GANT61 (Stemcell Technologies 73692), pasireotide (TargetMol TP2207), mifeprostone (Selleckchem S2606), etomidate (Selleckchem S1329), mitotane (Selleckchem S1732), metyropane (Selleckchem S5416), or osilodrostat (Selleckchem S7456) at concentrations of 0, 1, 10, 100, 1000, and 10,000 nM, for 72 h. The percentage of cell viability was measured using an MTS assay (Promega G3580). Absorbance was measured at 490 nm and normalized to the vehicle. Concentrations were plotted in a logarithmic scale, and a nonlinear dose response curve regression was calculated using GraphPad Prism. An IC50 value for each drug treatment was determined based on the dose response curve, using GraphPad Prism analysis software.

### 2.10. Calculation of Area under the Curve (AUC)

AUC (area under the curve) was determined by plotting the normalized % cell viability versus transformed concentration of the drugs, using a trapezoidal approximation for the area [31]. The formula was based on splitting the curve into trapezoids with bases equal to the % viability (*V*) and height equal to the interval length (difference in concentrations (*C*), and then summing the areas of each trapezoid:

$$\sum_{0}^{n}\frac{\left(V_{n}+V_{n-1}\right)}{2}*\left(C_{n}-C_{n-1}\right)$$

### 2.11. Quantitative RT PCR (qRT-PCR)

RNA was collected from patient-derived organoid cultures using the RNeasy Mini Kit (Qiagen). cDNA was generated from the extracted RNA, and then pre-amplified using TaqMan PreAmp Master Mix (Thermo Fisher Scientific 391128). The primers used were human-specific GAPDH (Thermo Fisher Scientific, Applied Biosystems Hs02786624_g1), NR5A1 (SF1) (Thermo Fisher Scientific, Hs00610436_m1), PIT1 (Thermo Fisher Scientific, Hs00230821_m1), TPit (Thermo Fisher Scientific, Hs00193027), and POMC (Thermo Fisher Scientific, Hs01596743_m1). Each PCR reaction was performed using a final volume of 20 µL, composed of 20X TaqMan Expression Assay primers, 2X TaqMan Universal Master Mix (Applied Biosystems, TaqMan^®^ Gene Expression Systems), and a cDNA template. Amplification of each PCR reaction was conducted in a StepOne™ Real-Time PCR System (Applied Biosystems, Foster City, CA, USA), using the following PCR conditions: 2 min at 50 °C, 10 min at 95 °C, denaturing for 15 s at 95 °C, and annealing/extending for 1 min at 60 °C, for a total of 40 cycles. Relative fold change was calculated using the 2 − ∆∆Ct method [32], where CT = threshold cycle. Results were analyzed as the average fold change in gene expression compared to the control, and GAPDH served as an internal control.

### 2.12. Whole Exome Sequencing

WES was performed by the University of Arizona Center for Applied Genetics and Genomic Medicine. Isolated DNA from patient adenoma tissue will be quantified using the Qubit quantitation system with standard curve, as per the supplier protocol (Thermo Fisher Scientific). All samples were further tested for quality using the Fragment Analyzer (Advanced Analytical), following the manufacturer-recommended protocols. Whole exome sequencing (WES) was performed by array capture and approximately 60 Mb of exome target sequence, using the SureSelectXT Human All Exon V6 enrichment (Agilent) or equivalent (which one was used). All exome library builds were quantified via qPCR and subsequently sequenced to a minimum 20X coverage, using paired-end chemistry on the Illumina NovaSeq platform. Whole exome sequencing (WES) was performed by hybridization capture of approx. 35 Mb of the exome target sequence, using the Swift Exome Hyb Panel (Swift Biosciences 83216). All exome library builds were quantified via qPCR and subsequently sequenced to a minimum 20X coverage, using paired-end chemistry on the Illumina NextSeq500 or NovaSeq platform (Illumina). DNA reads were trimmed, filtered by quality scores and aligned to the human genome (hg38) with Burrows–Wheeler Aligner with default parameters. Picard (http://broadinstitute.github.io/picard (accessed on 22 December 2021)) was used to mark duplicates. Germline single nucleotide variants (SNV) were called using the Genome Analysis Tool Kit (GATK), using the given guidelines. Mutations were annotated using ANNOVAR for coding sequences. Variants that passed the quality filter were further investigated for similarity. Concordance between tissue and organoids was calculated using Jaccard similarity index (J_ij_ = M_ij_/(M_i_ + M_j_ − M_ij_) where M_i_ is the number of variants in tissues, M_j_ is the number of variants in organoids, and M_ij_ is the number of identical variants in both tissue and organoid.

### 2.13. Single Cell RNA Sequencing (scRNA-Seq)

Cultures were collected on day 15 of the pituitary directed differentiation schedule, and cells were dissociated into a single-cell suspension using Cell Dissociation Buffer (Thermo Fisher Scientific 13151014). Cells (15,000 cells/sample) were resuspended in the sample buffer (BD Biosciences 65000062), filtered using cell strainer (40 microns), and loaded into a BD Rhapsody cartridge (BD Biosciences 400000847) for single-cell transcriptome isolation. Based on the BD Rhapsody system whole-transcriptome analysis for single-cell whole-transcriptome analysis, microbead-captured single-cell transcriptomes were used to prepare a cDNA library. Briefly, double-stranded cDNA was first generated from the microbead-captured single-cell transcriptome in several steps, including reverse transcription, second-strand synthesis, end preparation, adapter ligation, and whole-transcriptome amplification (WTA). Then, the final cDNA library was generated from double-stranded full-length cDNA by random priming amplification using a BD Rhapsody cDNA Kit (BD Biosciences, 633773), as well as the BD Rhapsody Targeted mRNA and WTA Amplification Kit (BD Biosciences, 633801). The library was sequenced in PE150 mode (paired-end with 150-bp reads) on NovaSeq6000 System (Illumina). A total of 80,000 reads were demultiplexed, trimmed, mapped to the GRCh38 annotation, and quantified using the whole transcriptome analysis pipeline (BD Rhapsody™ WTA Analysis Pipeline v1.10 rev6, San Jose, CA, USA) on the Seven Bridges Genomics platform (https://igor.sbgenomics.com (accessed on 4 April 2022)), prior to clustering analysis in Seurat. For QC and filtration, read counting and unique molecular identifier (UMI) counting were the principal gene expression quantification schemes used in this single-cell RNA-sequencing (scRNA-seq) analysis. The low-quality cells, empty droplets, cell doublets, or multiplets were excluded based on unique feature count (less than 200 or larger than 2500), as they may often exhibit either an aberrantly high gene count or very few genes. Additionally, the mitochondrial QC metrics were calculated, and the cells with >5% mitochondrial counts were filtered out, as the percentage of counts originating from a set of low-quality or dying cells often exhibit extensive mitochondrial contamination. After the removal of unwanted cells from the single cell dataset, the global-scaling normalization method LogNormalize was employed. This method normalizes the feature expression measurements for each cell by the total expression, multiplies this by a scale factor (10,000), and log-transforms the result. The molecules per gene per cell, based on RSEC error correction (RSEC_MolsPerCell file) matrix files from iPSC^ctrl^ and iPSC*^CDH23^* samples, were imported into Seurat v4, merged, and processed (as stated above) for UMAP reduction, cluster identification, and differential marker assessment using the FindAllMarkers function within Seurat.

### 2.14. Statistical Analyses

Sample size was based on assessment of power analysis using SigmaStat software. Data collected from each study from at least 4 in vitro technical replicates were analyzed by obtaining the mean ± standard error of the mean (SEM), unless otherwise stated. The significance of the results was then tested using commercially available software (GraphPad Prism, GraphPad software, San Diego, CA, USA).

## 3. Results

### 3.1. Generation and Validation of Human PitNET Tissue Derived Organoids

Human PitNET tissue was harvested during endoscopic transsphenoidal pituitary surgery from 35 patients in order to generate organoids. These cultures are referred to as human PitNET tissue derived organoids (hPITOs). Supplementary Table S3 summarizes the neuropathology reports and clinical diagnosis from these cases. In summary, 12 corticotroph (functional, CD), and 3 silent corticotroph tumors (nonfunctional tumors), 9 gonadotroph tumors, 8 lactotroph tumors, and 3 somatotroph tumors (acromegaly) were used to generate hPITOs (Supplementary Table S3).

**Figure 1:**
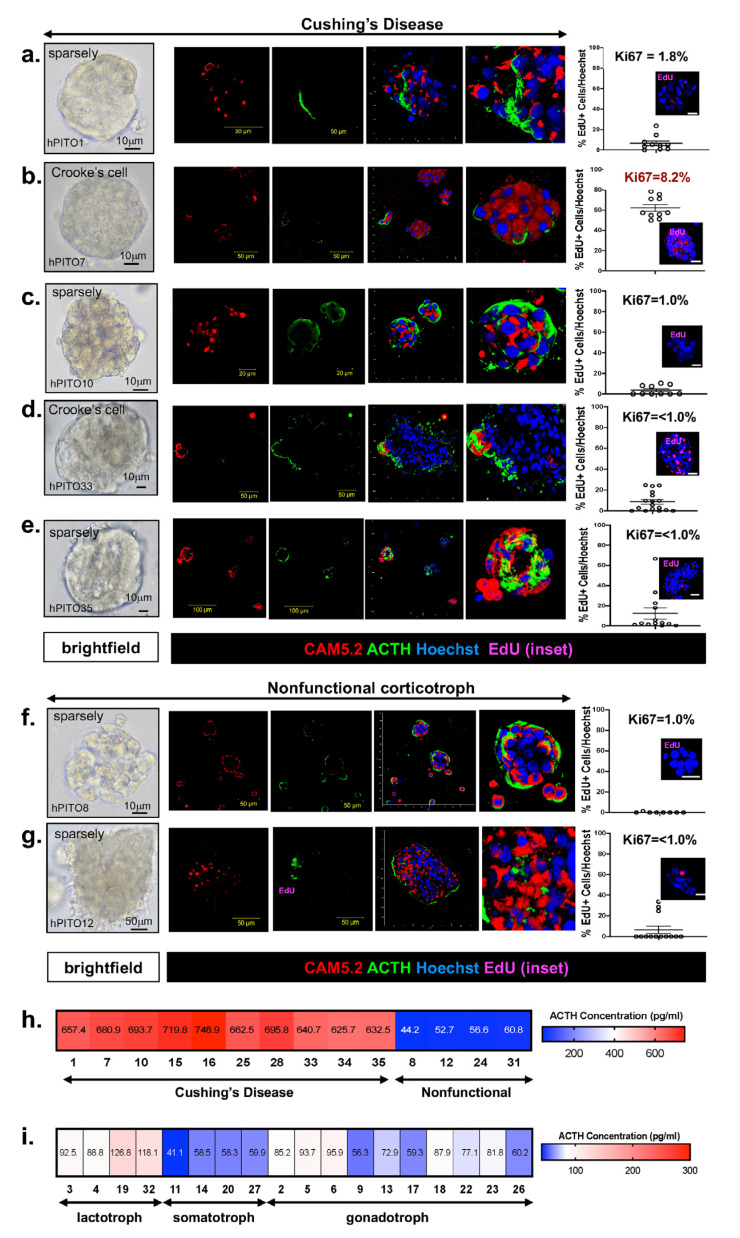
Morphology and function of corticotroph hPITOs. (**a**–**g**) Brightfield images, immunofluorescence staining using antibodies specific for CAM5.2 (red), ACTH (green), and EdU (magenta, inset) of organoid cultures generated from patients with Cushing’s disease (hPITOs 1, 7, 10, 33, 35) or nonfunctional corticotroph adenomas (hPITO8, 12). Quantification of %EdU positive cells/total cell number is shown and compared to the Ki67 score given in the pathology report (Supplemental Table S3). An ELISA was performed using conditioned media collected from (**h**) corticotroph hPITO cultures and (**i**) lactotroph, somatotroph, and gonadotroph hPITO cultures for the measurement of ACTH secretion (pg/mL).

Bright-field microscopy images of hPITOs that were generated from corticotroph adenomas from patients diagnosed with CD (Figure 1a–e). Silent/nonfunctioning tumors (Figure 1f,g) revealed morphological diversity among the organoid lines between individual patients and amongst subtypes. Confocal microscopy was used to capture a z-stack through the hPITO38, immunofluorescently stained for CAM5.2 (red), ACTH (green), and Hoechst (nuclear staining, blue) and emphasizes the 3D cellular structure of the hPITOs (Supplemental Video S1). Lactotroph, gonadotroph, and somatotroph adenomas were used to generate hPITOs, and showed the same morphological divergence amongst subtypes and between each patient line (Supplemental Figure S2). Proliferation was measured within the cultures using 5-ethynyl-2′-deoxyuridine (EdU) uptake and showed that the percentage of EdU+ve cells/total Hoechst+ve nuclei directly correlated with the pathology MIB-1 (Ki67) score (red, R^2^ = 0.9256) (Figure 1a–g, Supplemental Figure S2). ACTH concentration, which was measured by ELISA using organoid conditioned culture media collected from each hPITO line, showed the highest expression in the corticotroph adenoma organoids generated from CD patients (Figure 1h).

### 3.2. Characterization of Cell Lineages in Pituitary Adenoma-Derived Organoids by Spectral Cytek™ Aurora Analysis

**Figure 2:**
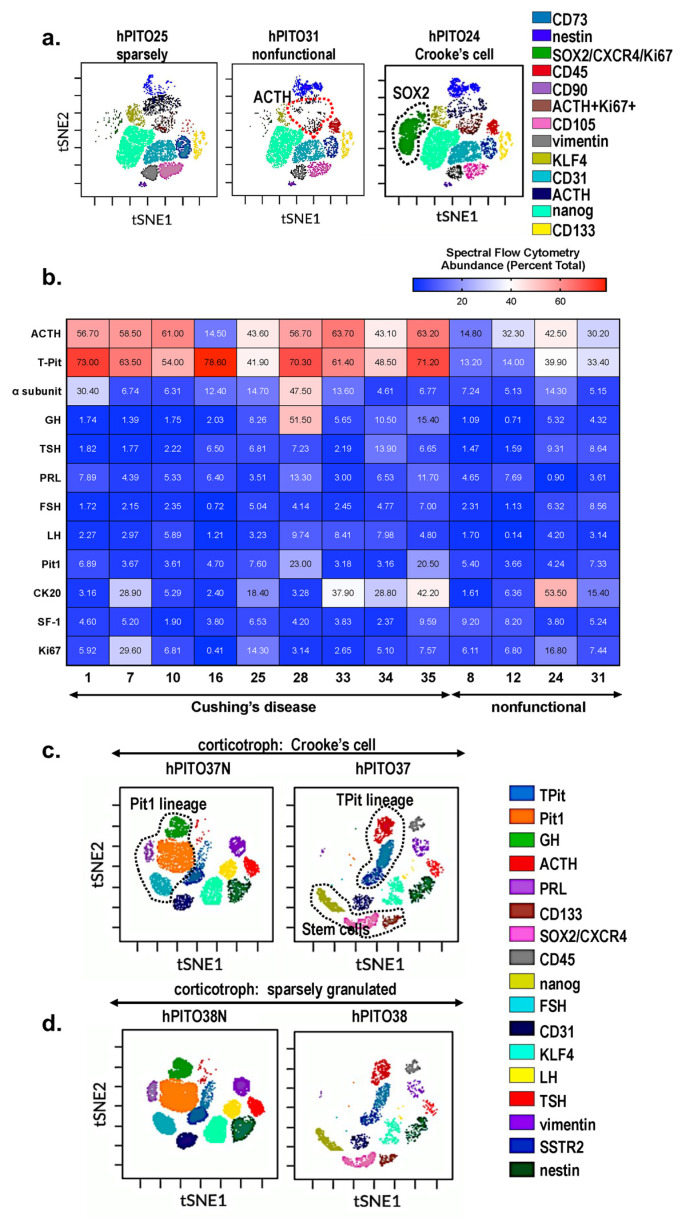
Cell heterogeneity of corticotroph hPITOs. (**a**) viSNE maps define spatially distinct cell populations using pituitary specific cell lineage, stem cell, and transcription factor markers. Cell populations were quantified in organoids generated from CD patients with corticotroph adenomas (sparsely granulated and Crooke’s cell adenoma) or patients with nonfunctional corticotroph adenomas. (**b**) Quantification of the abundance of cells expressing pituitary specific markers as a percent total. viSNE maps define spatially distinct cell populations in organoid cultures generated from CD patient with (**c**) corticotroph adenoma (hPITO37, Crooke’s cell adenoma) and adjacent normal tissue (hPITO37N), or (**d**) sparsely granulated corticotroph adenomas (hPITO38) and adjacent normal tissue (hPITO38N).

In order to validate the similarity in cell lineages identified between the organoid line and the patient’s tumor, we compared the immunohistochemistry from the neuropathology report (Supplemental Table S3) to the expression pattern of pituitary adenoma-specific markers, which were measured using Cytek™ Aurora spectral flow cytometry (Figure 2). The location of cells that are found in each cluster based on the highly expressed antigens are shown in the representative tSNE (viSNE) maps (Figure 2a). Compared to nonfunctional adenoma-derived hPITOs, organoids derived from corticotroph adenomas of CD patients highly expressed proliferating (Ki67+) T-Pit+ ACTH cells (Figure 2a). Interestingly, there was an increase in SOX2+ cells within the total cell population, associated with Crooke’s cell adenoma hPITOs (Figure 2a). Within the total cell population, cell clusters expressing CD45 and vimentin were also measured (Figure 2a). Data for the analysis of corticotroph hPITOs, derived from CD patients and individuals with nonfunctional adenomas, were summarized in a heatmap for each subtype organoid line based on quantified cell abundance (percent of total cells) using spectral flow cytometry (Figure 2b).

Organoid cultures derived from pituitary adenomas (hPITO37 and hPITO38) were compared to organoids derived from adjacent normal pituitary tissue (hPITO37N and hPITO38N) (Figure 2c,d). While Pit1 lineages including cells expressing GH and PRL, as well as SF1 lineages expressing FSH and LH, were detected in the hPITO37N and hPITO38N organoid cultures, these cell populations were significantly reduced within the patient’s matched adenoma tissue (Figure 2c,d). Overall, hPITOs derived from CD patients expressed increased stem and progenitor cell markers, including CXCR4, SOX2, and CD133 (Figure 2). Collectively, our findings of the characterization of the hPITO cultures support our prediction that this in vitro model recapitulates much of the patient’s adenoma pathophysiology.

### 3.3. Inherent Patient Differences to Drug Response Is Reflected in the Organoid Culture

**Figure 3:**
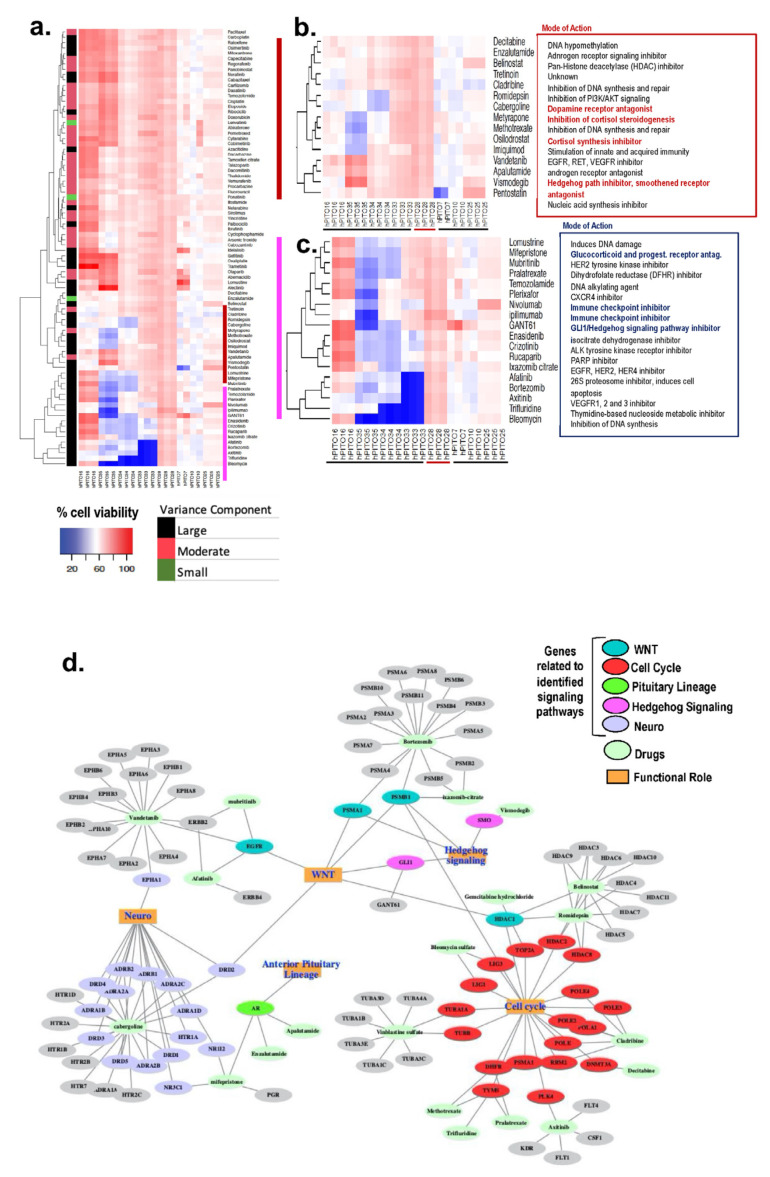
Drug screen using hPITOs generated from CD patients. (**a**) High-throughput drug screening of hPITOs reveals sensitivities to a range of therapeutic agents. Cell viability with high values (indicating resistance) are depicted in red, and low values (indicating sensitivity) are in blue in the clustered heatmap. (**b**,**c**) Clusters showing response to therapeutic agents with the most variance across the organoids. (**d**) Network of drugs from the clusters b and c and their gene targets, showing their participation in signaling pathways and cellular processes.

Tumor recurrence can occur in as many as 30–50% of CD patients after successful surgical treatment [10,33,34]. Unfortunately, bilateral adrenalectomy is the chosen surgical treatment for patients with persistent CD [35]. Bilateral adrenalectomy leads to the increased risk for development of Nelson’s syndrome (progressive hyperpigmentation due to ACTH secretion and expansion of the residual pituitary tumor). Although the risk of developing Nelson’s syndrome following adrenalectomy can be reduced by 50% with stereotactic radiotherapy [35], there is a need to develop medical therapies that directly target the pituitary adenoma. Thus, we established a high-throughput drug screening assay using patient-derived PitNET organoids. After 72 h of treatment, cell viability was measured using an MTS assay, and data were represented as a heatmap whereby blue indicated higher cell death, and red suggested higher cell viability. The replicates behaved consistently with the drug response, with correlation scores of >0.8 for these samples (Figure 3a). We estimated the variance component for each drug across all organoids. Variation among samples was found to be significant (*p* ≤ 0.05) for each of the 83 drugs. The drug responses were grouped by variance factor into large, median, and small. The larger the variance, the more variable the drug response was across the organoids. We noted a set of drugs that showed a significant differential response across the functional corticotroph organoids. Unsupervised clustering of drug responses across organoids shows a pattern that relates to our statistically calculated results (Figure 3a,c), and the replicates for each independent organoid cluster together. The drugs with higher variance components across all the functional corticotrophs cluster together as a group (Figure 3a). These drugs show cell viability of 10% to 60% across different organoids. Analyzing the pattern more closely, we observe that, within a pathologically defined group, there was a differential organoid response to drugs as well as inherent patient differences to drugs within this group. Figure 3 demonstrates a variation in drug responsiveness amongst the organoid lines generated from individual patients. Importantly, there was further divergence in drug responsiveness amongst the individual organoid lines within each pathologically defined corticotroph subtype. These data clearly demonstrate that the inherent patient difference to drug response which is often observed among CD patients is reflected in the organoid culture.

Drugs that clustered together and showed correlated responses were investigated further for their mode of action based on target genes (Figure 3d). The genes were analyzed for their associations in cellular pathways and gene ontology functional processes. Identified drug–gene pairs were interconnected by cellular pathways that are known to regulate cell cycle, WNT signaling, hedgehog signaling, and neuroactive ligand-receptor interaction signaling pathways (Figure 3d). These identified genes are also known to be influenced by multiple cellular functions, such as cytokine–cytokine receptor interactions and Notch signaling. Proteosome 20S subunit genes PSMAs/PSMBs and the HDAC gene family are involved in many cellular functions. The ephrin receptors (EPHs), adrenoceptor alpha receptors (ADRs), dopamine receptors (DRDs), and the 5-hydroxytryptamine serotonin receptors (HTRs) gene families influence neuronal functions and are targeted by multiple drugs in our focused cluster. These data reveal potential therapeutic pathways for CD patients.

**Figure 4:**
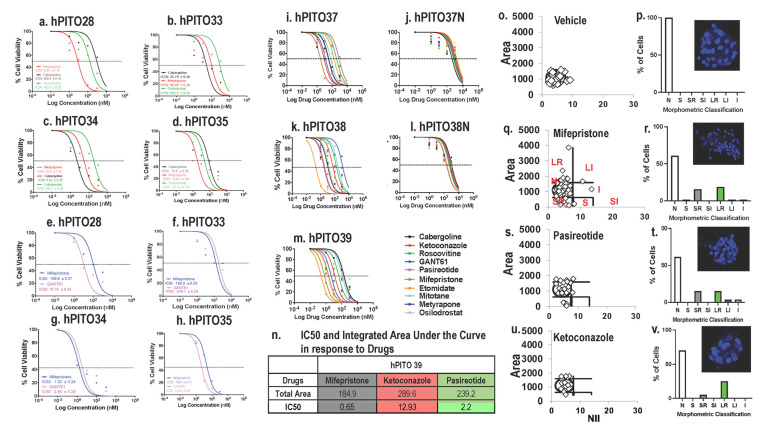
Drug dose responses by hPITOs generated from CD patients. Dose responses to mifepristone, GANT61, cabergoline, and osilodrostat. (**a**,**e**) hPITO28, (**b**,**f**) hPITO33, (**c**,**g**) hPITO34, and (**d**,**h**) hPITO35. Dose responses to cabergoline, ketoconazole, roscovitine, GANT61, pasireotide, mifepristone, etomidate, mitotane, metyrapone, and osilodrostat in (**i**) hPITO37, (**j**) organoids generated from adjacent normal pituitary tissue (hPITO37N), (**k**) hPITO38, (**l**) hPITO38N, and (**m**) hPITO39. (**n**) IC50 and integrated area under the curve in response to mifepristone, ketoconazole, and pasireotide using hPITO39 cultures. Nuclear morphometric analysis of hPITO39 cultures in response to (**o**,**p**) vehicle, (**q**,**r**) mifepristone, (**s**,**t**) pasireotide, and (**u**,**v**) ketoconazole. Morphometric classification of NII was based on the normal (N), small (S), small regular (SR), short irregular (SI), large regular (LR), large irregular (LI), and irregular (I) nuclear morphology. Representative Hoechst staining of organoids in response to drug treatments for the calculation of the nuclear irregularity index (NII) are shown in the insets in (**p**,**r**,**t**,**v**).

Divergent half maximal inhibitory concentration (IC50) values, as documented by an MTS cell viability assay, were observed in response to drug treatment among hPITOs lines 28, 33, 34, 35, and 37. Note that a shift of the curve to the right indicates a higher IC50 (i.e., more resistant to that drug). Cell viability assays were normalized to vehicle-treated controls in order to ensure that toxicity was specific to the drug effects (Figure 4). Dose response curves for organoid 33 and organoid 34 showed better responses at lower doses for cabergoline compared to Metyrapone and osilodrostat, but different for organoid 35, where Metyrapone and osilodrostat gave better responses than Cabergoline (Figure 4a–h). For the drugs mifepristone and GANT61, 33 and 34 had the same level of response to both the drugs. However, when the two organoid responses were compared, 34 had a better response than 33 (Figure 4a–h). Similar divergent drug responses were observed in hPITO lines 37 and 38 (Figure 4i,k). However, organoids generated from adjacent normal pituitary tissue from patients 37 and 38 were nonresponsive to the same standard of care of investigational drugs for CD (Figure 4j,l). These data were consistent with observation made in the drug screen (Figure 3a–c), and demonstrate that there was an inherent difference to drug response within the organoid cultures of the same corticotroph subtype.

In addition to cell viability, Nuclear Morphometric Analysis (NMA) using treated organoids was performed based on a published protocol that measures cell viability according to the changes in nuclear morphology of the cells, using nuclear stain Hoechst or DAPI [30]. Nuclear Irregularity Index (NII) was measured based on the quantification of the morphometric changes in the nuclei in response to the standard-of-care drugs mifepristone, pasireotide, and ketoconazole in hPITO39 (Figure 4o–v). The area vs. NII of vehicle-treated cells were plotted as a scatter plot using the template, and considered as the normal cell nuclei (Figure 4o). The same plots were generated for mifepristone (Figure 4q), pasireotide (Figure 4s), and ketoconazole (Figure 4u). The NII and area of treated cells were compared to those of the normal nuclei, and classified as one of the following NMA populations: Normal (N; similar area and NII), Mitotic (S; similar area, slightly higher NII), Irregular (I; similar area, high NII), Small Regular (SR; apoptotic, low area and NII), Senescent (LR; high area, low NII), Small Irregular (SI; low area, high NII), or Large Irregular (LI; high area, high NII) (Figure 4p,r,t,v). Cells classified as SR exhibited early stages of apoptosis, and cells classified as either I, SI, or LI exhibited significant nuclear damage. Data showed that mifepristone induced significant apoptosis in hPITO39 cultures (Figure 4r), compared to responses to pasireotide (Figure 4t) and ketoconazole (Figure 4v). These responses were consistent with the IC50 and the total area under the curve in response to drugs (Figure 4m,n). Measurement of NII is an approach which may be used to confirm potential drug targets identified from the drug screen.

### 3.4. Organoid Responsiveness to Pasireotide Correlates with SSTR2 and SSTR5 Expression

**Figure 5:**
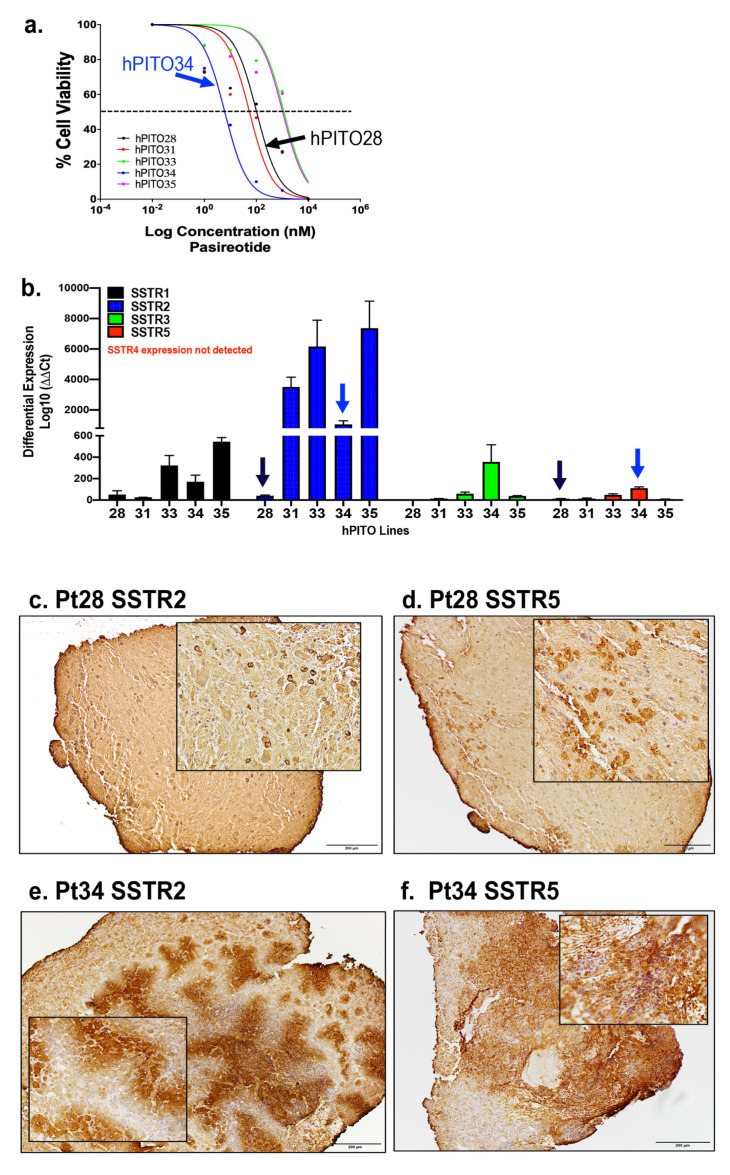
SSTR1-5 expression in hPITOs and patient’s PitNET tissue. (**a**) Dose response of hPITO28, 31, 33, 34, and 35 lines to pasireotide. (**b**) Differential expression of SSTR subtypes 1–5 (SSTR1, SSTR2, SSTR3, SSTR4, SSTR5) in hPITO28, hPITO31, hPITO33, hPITO34, and hPITO35. Immunohistochemistry of (**c**,**e**) SSTR2 and (**d**,**f**) SSTR5 expression in patient PitNET tissue (Pt28 and Pt34), from which hPITO28 and 34 were generated.

Organoid lines hPITO28, 31, 33, 34, and 35 exhibited divergent IC50 values in response to SSTR agonist pasireotide (Figure 5a). hPITO34 was the most responsive to pasireotide, with a low IC50 value of 6.1 nM (Figure 5a). Organoid lines hPITO33 and hPITO35 were the least responsive, with IC50 values of 1.2 µM and 1 µM, respectively, in response to pasireotide (Figure 5a). The expression of SSTR subtypes 1–5 among the different organoid lines were measured by qRT-PCR and IHC (Figure 5b). One of the least responsive organoid lines, hPITO28, exhibited lower differential expression in SSTR2 and SSTR5 compared to the highly responsive hPITO34 line (Figure 5a,b). Gene expression levels of SSTR2 and SSTR5 within hPITO28 and 34 correlated with protein levels within the patient’s tumor tissue (Figure 5c–f). Given the greater binding affinity for SSTR5 compared to SSTR2 by pasireotide, these data were consistent with greater responsiveness to the drug by hPITO34 in comparison to hPITO28 (Figure 5a,c–f). The expression of SSTR subtypes 2 and 5 within the organoid cultures correlated with the expression patterns of the patient’s tumor tissues (Figure 5a,c–f).

### 3.5. Organoids Derived from Pituitary Corticotroph Adenomas Retain the Genetic Alterations of the Patient’s Primary Tumor

**Figure 6:**
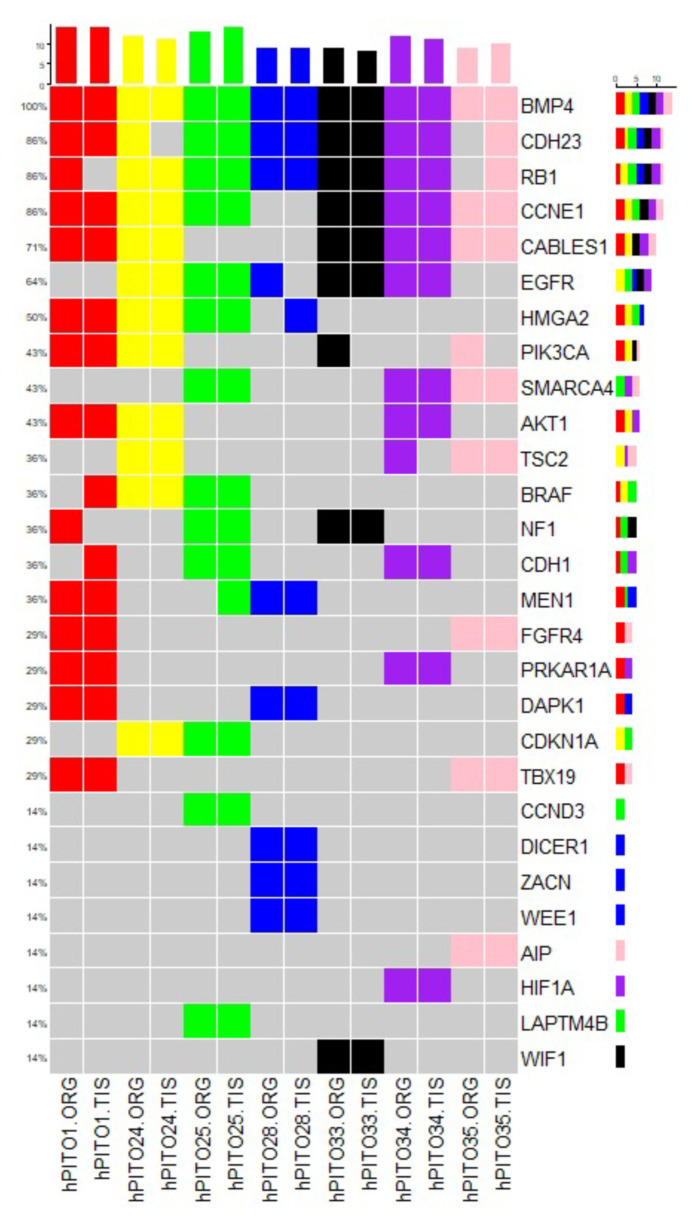
Genomic landscape of hPITOs recapitulates genetic alterations commonly found PitNETs. Overview of single nucleotide variation events detected in hPITOs in genes commonly altered in PitNETs. The mutation frequency across the organoid population is depicted on the right. Color coding of the figure shows that organoid lines are derived from the same patient tumor tissue. ORG: organoid line, TIS: matched patient’s PitNET tissue.

In order to identify the genetic features of the organoids derived from pituitary adenomas of CD patients, we performed whole-exome sequencing (WES) of hPITOs and the corresponding primary adenoma tissues. We performed WES analysis of each hPITO line, and compared the results with those for the corresponding primary adenoma tissues. We showed the concordance rate of exonic variants between the primary tumor tissues obtained from CD patients and the corresponding organoid line. We identified, on average, approximately 5000 mutations across each of the 14 paired samples of organoids and tissues. For the variants detected, all seven pairs showed a Jaccard index ranging from 0.5 to 0.8. Out of seven pairs, five (hPITO24, 25, 28 and 35) pairs had a Jaccard score of 0.8, while hPITO33 and 34 pairs had 0.7, and hPITO1 had 0.5. In order to investigate the similarity across the SNV (single nucleotide variation) sites, we calculated the Jaccard index of exon sites for synonymous and non-synonymous events, and found scores for all pairs ranging from 0.8 to 0.9. Furthermore, for only non-synonymous events, Jaccard scores also ranged from 0.8 to 0.9, except for hPITO1, which showed overall lower concordance, and had a score of 0.4 to 0.5. Figure 6 shows non-synonymous mutations found in organoid and tissue pairs for some of the key genes that are known to be involved in pituitary adenoma disease. Concordance indices between organoids and the matched patient’s adenoma tissues is reported in Figure 6. Therefore, WES data demonstrated that organoids derived from pituitary corticotroph adenomas retained the genetic alterations of the patient’s primary tumor tissue.

### 3.6. IPSC Pituitary Organoids Generated from a CD Patients Expressing Familial Mutations Reveal Corticotroph Adenoma Pathology In Vitro

Extensive research has revealed the role of somatic and germline mutations in the development of CD adenomas [36,37]. Pituitary organoids were developed from iPSCs generated from the PBMCs of CD patients and carrying germline mutations that were identified by WES (Supplemental Figure S4). Chromosomal aberrations were not found when comparing against the reference dataset in the iPSCs generated from the CD patients (Supplemental Figure S3a,b). PBMCs isolated from patients diagnosed with CD were analyzed by WES in order to determine the expression of germline mutations. WES revealed the expression of a more recently identified gene predisposing patients to CD, namely cadherin-related 23 [38] (Supplemental Figure S5).

Pituitary organoids were then developed from iPSCs which were generated from the PBMCs of patients with CD (iPSC*^CDH23^* and iPSC*^MEN1^*) and a healthy individual (iPSC^ctrl^). Expression of PIT1 (pituitary-specific positive transcription factor 1), ACTH (adrenocorticotropic hormone), GH (growth hormone), FSH (follicle-stimulating hormone), LH (luteinizing hormone), PRL (prolactin), and synaptophysin (synaptophysin) with co-stain Hoechst (nuclei, blue) was measured by immunofluorescence, using chamber slides collected at 15 of the differentiation schedules (Supplemental Figure S6). While pituitary tissue that was differentiated from iPSC^ctrl^ expressed all major hormone-producing cell lineages (Supplemental Figure S6a), there was a significant increase in the expression of ACTH and synaptophysin, with a concomitant loss of PIT1, GH, FSH, LH, and PRL in iPSCs*^MEN1^* (Supplemental Figure S6b,c). Interestingly, iPSC*^CDH23^* cultures exhibited a significant increase in the expression of ACTH, GH, LH, and synaptophysin, with a concomitant loss of PIT1, FSH, and PRL (Supplemental Figure S6b,c). Immunofluorescence of iPSCs collected on the fourth day of the differentiation schedule revealed no expression of PIT1, ACTH, GH, FSH, LH, or PRL in (data not shown). Compared to control lines, iPSC lines expressing mutated *CDH23* secreted significantly greater concentrations of ACTH earlier in the differentiation schedule (Supplemental Figure S7a). The upregulated expression of pituitary corticotroph adenoma-specific markers in iPSC*^CDH23^* and iPSC*^MEN1^* demonstrates that the iPSC-derived organoids represented the pathology of corticotroph adenomas in vitro.

### 3.7. ScRNA-seq Reveals the Existence of Unique Proliferative Cell Populations in iPSC^CDH23^ Cultures When Compared to iPSCs^ctrl^

**Figure 7:**
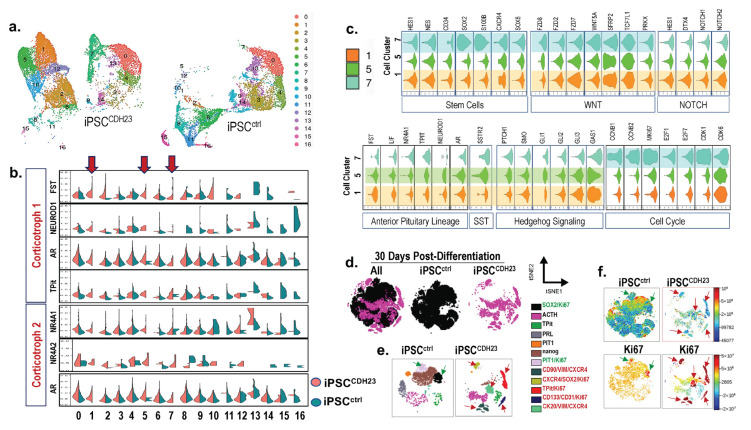
Single cell analysis of iPSC^ctrl^ and iPSC*^CDH23^* cultures 15 and 30 days post-directed differentiation. (**a**) UMAP plots showing identified cell clusters 0–16 in iPSC^ctrl^ and iPSC*^CDH23^* cultures 15 days post-directed differentiation. (**b**) Violin plots of representative identified markers of the corticotroph cell lineage, where 2 subpopulations were observed among iPSC^ctrl^ and iPSC*^CDH23^* cultures. Arrows highlight clusters 1, 5, and 7. (**c**) Violin plots showing expression of genes representative of stem cells, Wnt, NOTCH, Hh and SST signaling, anterior pituitary (corticotroph) cell lineage, and cell cycle in clusters 1, 5, and 7 of iPSC*^CDH23^* cultures. Plot width: cell number, plot height: gene expression. (**d**) viSNE maps showing concatenated flow cytometry standard files for both samples and iPSC^ctrl^ and iPSC*^CDH23^* organoids 30 days post-directed differentiation. (**e**) Overlay of manually gated cell populations onto viSNE plots. (**f**) Fluorescent intensity of Ki67 of viSNE maps for both samples and iPSC^ctrl^ and iPSC*^CDH23^* organoids. iPSC^ctrl^ = 22518 events; iPSC*^CDH23^* = 17542 events.

Using Seurat to identify cell clusters, as well as Uniform Manifold Approximation and Projection 9UMAP, clustering analysis identified 16 distinct cell populations/clusters consisting of known marker genes. Clusters 1, 5, and 7 of the iPSCs*^CDH23^* were distinct from the iPSC*^ctrl^* cultures (Figure 7a,b). Pituitary stem cells were characterized in iPSC*^ctrl^* and iPSC*^CDH23^* cultures (Figure 7b). Clusters 1 and 5 expressed markers consistent with the corticotroph subtype cell lineage (Figure 5c). Markers of dysregulated cell cycles and increased proliferation were identified in cell cluster 7 (Figure 7c). Expression of the E2 factor (E2F) family of transcription factors, which are downstream effectors of the retinoblastoma (RB) protein pathway and play a crucial role in cell division control, were identified in distinct cell cluster 7, which was identified within the iPSC*^CDH23^* cultures (Figure 7c). Stem cell markers were also upregulated in cell cluster 7, and identified within the iPSC*^CDH23^* cultures (Figure 7c). Using Cytobank software to analyze organoids collected 30 days post-differentiation, cells were gated on live CK20 positive singlets, and 9000 events per sample were analyzed by the viSNE algorithm. ViSNE plots are shown in two dimensions with axes identified by tSNE- 1 and tSNE-2, and each dot representing a single cell positioned in the multidimensional space (Figure 7d). Individual flow cytometry standard files were concatenated into single flow cytometry standard files, from which 12 spatially distinct populations were identified (Figure 7e). Overlaying cell populations identified by traditional gating strategies onto viSNE plots identified unique cell populations within the iPSC*^CDH23^* cultures (Figure 7e). There were distinct cell populations between the iPSC*^ctrl^* and iPSC*^CDH23^* organoids, in addition to expression of hormone and cell lineage markers such as ACTH, TPit, PRL, and PIT1 (Figure 7e). The cell populations that exhibited high expression of Ki67 within the iPSC*^ctrl^* organoid cultures included SOX2+ and PIT1+ populations (Figure 7f). The highly proliferating cell populations within the iPSC*^CDH23^* organoid cultures included those that expressed CD90+/VIM+/CXCR4+ (mesenchymal stem cells), CXCR4+/SOX2+ (stem cells), TPit+ (corticotroph cell lineage), CD133+/CD31+ (endothelial progenitor cells), and CK20+/VIM+/CXCR4+ (hybrid epithelial-mesenchymal stem cells) (Figure 7f). Overall, the iPSC*^CDH23^* organoids were significantly more proliferative compared to the iPSC*^ctrl^* cultures (Figure 7f). Immunofluorescence staining of iPSC*^CDH23^* organoids revealed increased mRNA expression of TPit and POMC, which correlated with increased ACTH protein compared to iPSCs*^ctrl^* (Supplemental Figure S6). As shown in Supplemental Figure S6b,c, iPSC*^CDH23^* cultures also exhibited a significant increase in the expression of GH and LH (Supplemental Figure S6b,c).

Collectively, Figure 7 demonstrates that the development of pituitary organoids generated from iPSCs of CD patients may reveal the existence of cell populations which, potentially, contribute to the support of adenoma growth and progression, as well as an expansion of stem and progenitor cells that may be the targets for tumor recurrence.

## 4. Discussion

Our studies demonstrate the development of organoids generated from human PitNETs (hPITOs) can potentially be used to screen for the sensitivity and efficacy of responses to targeted therapies for CD patients that either fail to achieve remission or exhibit recurrence of disease after surgery. In addition, we have documented that induced pluripotent stem cells (iPSCs) generated from a CD patient expressing germline mutation CDH23 (iPSC*^CDH23^*) reveals the disease pathogenesis under directed differentiation. Many early in vitro experiments have used pituitary cell lines, spheroids, aggregates, and/or tumoroids that do not replicate the primary PitNET microenvironment [19,20,21], and lack a multicellular identity [39,40]. The development of PitNET tissue-generated organoids is limited to the use of transgenic mouse models as the source [22,23,41]. The recent organoid cultures reported by Nys et al. [42] have been generated from single stem cells isolated from PitNET tissue, and are claimed to be true organoids due to their clonality. However, multicellular complexity was not validated by the protein expression or hormone secretion from pituitary cell lineages in these cultures [42]. According to the National Cancer Institute (NCI, NIH), an ‘organoid’ is defined as “a tiny, 3-dimensional mass of tissue that is made by growing stem cells (cells from which other types of cells develop) in the laboratory” [43]. The hPITOs reported here begin from single and/or 3–4 cell clusters dissociated from the PitNET tissue that harbors the stem cells. Supplemental Video S2 demonstrates a process of ‘budding,’ as well as lumen formation as organoids grow and differentiate. We document differentiation and function by comprehensive spectral flow cytometry, ELISA, and response to standard of care drugs. The growth of PitNET organoids reported in the current study is consistent with that of gastrointestinal tissue derived cultures that begin from cell clusters, crypts, or glands [27,44,45].

Our studies report a PitNET tissue organoid culture with a multicellular identity consisting of differentiated cell lineages, stem/progenitor cells, and immune and stromal cell compartments, which replicates much of the patient’s own adenoma pathology, functionality, and complexity. We have also demonstrated that iPSCs, derived from the blood of a CD patient, can be directly differentiated into pituitary organoids that resemble similar characteristics to the tumor tissue. Many investigators have proposed the use of organoids in personalized medicine, but have focused these efforts on targeted treatment of cancers [27,46,47,48]. The findings reported in these studies are the first to implement this approach for the potential treatment of PitNETs. Collectively, we have developed a relevant human in vitro approach to potentially advance our knowledge as well as our approach to studies in the field of pituitary tumor research. Both the hPITOs and the iPSC*^CDH23^* may be implemented in studies that strive to (1) define the molecular and cellular events that are crucial for the development of PitNETs leading to CD, and (2) accelerate the identification of effective targeted therapies for patients with CD.

While published studies have advanced our understanding of the molecular mechanisms of the pathogenesis of corticotroph adenomas and elucidated candidate therapeutic targets for CD, these reports fall short of directly informing clinical decisions for patient treatment. Using organoids to screen potential drugs and compounds can potentially improve therapeutic accuracy. Figure 3 demonstrated a variation in drug responsiveness amongst the organoid lines generated from individual patients. Importantly, there was further divergence in drug responsiveness amongst the individual organoid lines within each pathologically defined corticotroph subtype. For example, hPITOs generated from patients with sparsely granulated corticotroph adenomas (hPIT0s 10, 25, 34, 35) and Crooke’s cell adenomas (hPITOs 7, 33) showed variable responses regardless of similar pathologically defined subtypes. In addition*,* the response of the tumor cells within the organoids to the standard of care drugs that directly target the pituitary in the body, including mifepristone and cabergoline, was only 50% in hPITO34 and hPITO35, and almost 0% in the other lines, including hPITO7, 10, and 25. These data clearly demonstrate that the inherent patient difference to drug response that is often observed among CD patients is reflected in the organoid culture. This culture system may be an approach that will provide functional data revealing actionable treatment options for each patient. Patient-derived organoids from several tumors have served as a platform for testing the efficacy of anticancer drugs and predicting responses to targeted therapies in individual patients [27,46,48,49,50]. An example of the use of organoids in identifying drug responsiveness within an endocrine gland is that of papillary thyroid cancer [51]. Organoids developed from PTC patients were used as a preclinical model for studying responsiveness to anticancer drugs in a personalized approach [51]. However, our study is the first report of the use of hPITOs for drug screening. Connecting genetic and drug sensitivity data will further categorize corticotroph subtypes associated with CD. WES analysis of each hPITO line was compared to the results for the corresponding primary adenoma tissues. We showed the concordance rate of exonic variants between the primary tumor tissues obtained from CD patients and the corresponding organoid line. On average, approximately 80% of the variants observed in the CD patients’ adenoma tissues were retained in the corresponding hPITOs.

Pituitary organoids were also developed from iPSCs generated from PBMCs of a CD patient expressing a germline genetic alteration in cadherin-related 23 *CDH23* (iPSC*^CDH23^*), a CD patient expressing an MEN1 mutation (iPSC*^MEN1^*), and a healthy individual (iPSC^ctrl^). Foundational studies performed by investigators at the genome level have revealed significant knowledge regarding the pathophysiology of CD [36,37,52,53]. In some instances, CD is a manifestation of genetic mutation syndromes that include multiple endocrine neoplasia type 1 (MEN1), familial isolated pituitary adenoma (FIPA), and Carney complex [54,55]. *CDH23* syndrome is clinically associated with the development of Usher syndrome, deafness, and vestibular dysfunction [56]. Several mutations in *CDH23* are associated with inherited hearing loss and blindness [57]. However, none of the variants found in this study were linked to any symptoms of deafness or blindness. A possible explanation is that deafness-related *CDH23* mutations are caused by either homozygous or compound heterozygous mutations [57]. In a study that linked mutations in *CDH23* with familial and sporadic pituitary adenomas, it was suggested that these genetic alterations could play important roles in the pathogenesis of CD [38]. Genomic screening in a total of 12 families with familial PitNETs, 125 individuals with sporadic pituitary tumors, and 260 control individuals showed that 33% of the families with familial pituitary tumors and 12% of individuals with sporadic pituitary tumors expressed functional or pathogenic *CDH23* variants [38]. Consistent with the expected pathology and function of a PitNET from a patient with CD, iPSC*^CDH23^* organoids exhibited hypersecretion of ACTH, and expression of transcription factors and cell markers were reported in the pathology report for corticotroph PitNETs. Collectively, these findings warrant further investigation to determine whether carriers of *CDH23* mutations are at a high risk of developing CD and/or hearing loss. Specifically, clinical investigation is required to determine whether pituitary MRI scans should be adopted in the screening of *CDH23*-related diseases, including Usher syndrome and age-related hearing loss.

Pituitary organoids generated from iPSCs of a CD patient revealed the existence of cell populations that potentially contribute to the support of PitNET growth and disease progression, as well as an expansion of stem and progenitor cells that may be the targets for tumor recurrence. Organoids derived from both pituitary adenomas and iPSCs exhibited increased expression of stem cell and progenitor markers at both the protein and transcriptomic levels. Unique clusters that were proliferative in the iPSC*^CDH23^* organoids expressed a hybrid pituitary cell population which was in an epithelial/mesenchymal state (CK20+/VIM+/CXCR4+/Ki67+). In support of our findings, a similar report of a hybrid epithelial/mesenchymal pituitary cell has been made as part of the normal developmental stages of the human fetal pituitary [58]. Previous studies have suggested that pituitary stem cells undergo an EMT-like process during cell migration and differentiation [59,60,61]. Consistent with our findings are extensive studies using single cells isolated from human pituitary adenomas to show increased expression of stem cell markers SOX2 and CXCR4 [22,23,41,62,63]. Within the clusters identified in the iPSC*^CDH23^* culture were cell populations expressing stem cell markers, including SOX2, NESTIN, CXCR4, KLF4, and CD34. The same iPSC*^CDH23^* cell clusters, 4, 8, 9, and 11, co-expressed upregulated genes of NOTCH, Hedgehog, WNT, and TGFβ signaling, which are pivotal not only in pituitary tumorigenesis and pituitary embryonic development, but also in ‘tumor stemness’ [22,23,41,62,63,64]. We also noted that clusters of cell populations 5 and 14 unique within the iPSC*^CDH23^* cultures expressed upregulated genes which were indicative of high proliferation. We observed upregulated expression of the E2F family of transcription factors (E2Fs) E2F1 and E2F7. These findings are of significance, given that there is evidence to show that upregulation of E2Fs is fundamental for tumorigenesis, metastasis, drug resistance, and recurrence [65]. Within the pituitary adenoma microenvironment, whether these stem cells directly differentiate into pituitary tumors or support the growth of the adenoma is largely unknown. In addition, whether pituitary stem cell populations become activated in response to injury is also understudied. Although the role of stem cells has been identified using a mouse model through implantation of the cells within the right forebrain [66], the identification of pituitary tumor-initiating stem cells using in vivo orthotopic transplantation models is impossible in mice. Pituitary tumors harboring the stem cells may require engraftment within the environment from which the cells are derived in order to enable growth and differentiation of the tumor. However, it is technically impossible to implant cells orthotopically in the murine pituitary. The pituitary tumor organoid cultures presented in these studies may offer an approach by which isolation, identification, and characterization of this stem cell population is possible. Therefore, we would gain knowledge on the mechanisms of pituitary tumor pathogenesis and reveal potential novel targets for therapeutic interventions by using the iPSC generated pituitary organoid culture.

PitNETs associated with the development of CD cause serious morbidity due to chronic cortisol exposure that dysregulates almost every organ system in the body. Overall, existing medical therapies remain suboptimal, with negative impact on health and quality of life, including considerable risk of therapy resistance and tumor recurrence. To date, little is known about the pathogenesis of PitNETs. Here, we present a human organoid-based approach that will allow us to acquire knowledge of the mechanisms underlying pituitary tumorigenesis. Such an approach is essential to identify targeted treatments and improve clinical management of patients with CD.

## 5. Conclusions

Cushing’s disease (CD) is a serious endocrine disorder caused by an adrenocorticotropic hormone (ACTH)-secreting pituitary neuroendocrine tumor (PitNET), which stimulates the adrenal glands to overproduce cortisol. The absence of preclinical models that replicate the PitNET microenvironment has prevented us from acquiring the knowledge to identify therapies that can be targeted to the tumor with a higher efficacy and tolerability for patients. Our studies demonstrate the development of organoids generated from human PitNETs or induced pluripotent stem cells as an essential approach to identifying targeted therapy methods for CD patients.

## Data Availability

The datasets generated during the analysis of the present study are available in the ReDATA repository, https://doi.org/10.25422/azu.data.19755244.v1. The datasets generated in the current study are also available from the corresponding author on reasonable request. All data generated or analyzed during this study are included in this published article (and its Supplementary Information Files).

## Supplementary Materials

The following supporting information can be downloaded at: https://www.mdpi.com/article/10.3390/cells11213344/s1, Figure S1: Antibodies used and Cytek^®^ Full Spectrum Viewer showing calculated similarity indices; Figure S2: Morphology and proliferation of lactotroph, somatotroph, and gonadotroph hPITOs; Table S1: Pituitary Growth Media; Table S2: Components used for pituitary organoids generated from iPSCs; Table S3: clinical characteristics of pituitary adenoma samples used for the generation of organoids; Table S4: Average correlation of replicates reported in Figure 3; Table S5: pituitary cell lineage or stem cell markers used in the scRNA-seq analysis; Video S1: hPITO38 EdU ACTH 3.
