# Supplementary material for: Development of Human Pituitary Neuroendocrine Tumor Organoids to Facilitate Effective Targeted Treatments of Cushing’s Disease
Source: Cells. 2022 Oct 23;11(21):3344. doi: 10.3390/cells11213344 (PMC9659185; doi:10.3390/cells11213344)
Supplement: Supplementary file 1 [file cells-11-03344-s001.zip › cells-1952672-Supplementary Figures.pdf]

Supplementary Figures

Marker	Fluorophore	Antibodies	Secondary Antibodies
ACTH	AF532	Novus Biological # NBP2-34529AF532	-
LH beta Antibody	DyLight 405	Novus Biological # NBP2-47733V	-
PRL	PE Cy7	Novus Biological # NBP2-73609PECY7	-
FSH	AF350	Novus Biological # NBP2-47735AF350	-
GH	AF750	Novus Biological # NBP2 54352AF750	-
TPit1	DyLight 550	Novus Biological # NBP2 74469C	
SF1	AF594	Novus Biological # 74338AF594	
TSH	BV 605	Novus Biological # NBP2-52574	Biolegend # 405327 anti Mouse
CK20	AF700	Novus Biological # NBP2-42816AF700	
Panck	DyLight 755	Novus Biological # NBP2-33200IR	-
Alpha hcg	BV480	Novus Biologicals # NBP3-11024 -	Jackson Immuno research # 109-685-170 (Goat IgG anti-Human IgG)
Ki67	BV711	Biolegend # 350515	
SOX2	PacificBlue	Biolegend # 656111	
Nestin	APC	Thermofisher # MA5-23650	
CD133	PEDazel 594	Biolegend # 141211	
Vimentin	Percp/Cy5.5	-Novus Biological # MAB 2105	Biolegend # anti Rat
CXCR4	PE/Cy5	Biolegend # 3066507	
CD73	BUV737	BD Biosciences # 612812	
CD90	BUV496	BD BioSciences # 741160	
CD105	BUV661	BD BioSciences # 750036	
KLF4	Dylight 550	Novus Biological # NBP1-21047R	
Pit1	PE/AF 610	ThermoFisher # PA5 98650	Thermo Fisher # A20961 Anti Rabbit
Nanog	AF555	BD Biosciences # 560260	
CD31	BV510	BD Biosciences # 563454	
CD45	BV786	BD Biosciences # 684555	
SSTR5	FITC	Novus Biological # FAB4448F	
SSTR2	Percp	Novus Biological # FAB4224C	

Similarity™ Indices

Configuration:5L 16UV-16V-14B-10YG-8R

	Alexa Fluor 532	DyLight 405	PE-Cy7	Alexa Fluor 350	Alexa Fluor 750	Alexa Fluor 594	Alexa Fluor 700	Alexa Fluor 555	DyLight 650	DyLight 550	DyLight 800	BV605	BV480	BV711	BV510	BV786	BUV737	BUV496	BUV661	PE-Cy5	PE-Alexa Fluor 610	PE-Dazzle594	PerCP-Cy5.5	PerCP	Pacific Blue	FITC	APC
Alexa Fluor 532	1																										
DyLight 405	0.02	1																									
PE-Cy7	0.01	0	1																								
Alexa Fluor 350	0.02	0.16	0.02	1																							
Alexa Fluor 750	0	0	0.24	0.02	1																						
Alexa Fluor 594	0.06	0	0.09	0.04	0.05	1																					
Alexa Fluor 700	0	0	0.09	0.01	0.4	0.06	1																				
Alexa Fluor 555	0.32	0.09	0.03	0.37	0.02	0.44	0	1																			
DyLight 650	0	0	0.05	0.01	0.36	0.11	0.66	0.02	1																		
DyLight 550	0.22	0	0.04	0.01	0.03	0.51	0.02	0.81	0.03	1																	
DyLight 800	0	0	0.18	0.02	0.81	0.01	0.23	0	0.17	0	1																
BV605	0.04	0.06	0.03	0.13	0.03	0.59	0.03	0.38	0.03	0.23	0.01	1															
BV480	0.01	0.27	0	0.25	0	0.04	0.01	0.34	0	0	0	0.16	1														
BV711	0	0.09	0.11	0.08	0.25	0.1	0.44	0.04	0.22	0.01	0.17	0.17	0.03	1													
BV510	0.02	0.15	0	0.32	0.01	0.1	0.01	0.46	0	0	0	0.4	0.84	0.06	1												
BV786	0	0.19	0.17	0.08	0.3	0.05	0.11	0.03	0.02	0	0.28	0.09	0.05	0.54	0.06	1											
BUV737	0	0	0.14	0.05	0.37	0.04	0.51	0.02	0.29	0	0.25	0.05	0	0.41	0.02	0.29	1										
BUV496	0.02	0.06	0	0.69	0	0.01	0.01	0.46	0	0	0	0.05	0.38	0	0.48	0.01	0.01	1									
BUV661	0	0	0.05	0.06	0.2	0.23	0.42	0.08	0.7	0.07	0.08	0.17	0.01	0.25	0.04	0.07	0.37	0.02	1								
PE-Cy5	0.06	0	0.14	0.02	0.09	0.54	0.2	0.19	0.35	0.23	0.03	0.21	0	0.14	0.01	0.03	0.1	0	0.42	1							
PE-Alexa Fluor 610	0.13	0	0.11	0.03	0.05	0.84	0.1	0.36	0.16	0.42	0.01	0.4	0.01	0.11	0.04	0.03	0.06	0	0.29	0.82	1						
PE-Dazzle594	0.3	0	0.05	0.04	0.04	0.84	0.03	0.67	0.06	0.75	0	0.47	0.04	0.04	0.09	0.01	0.02	0.02	0.13	0.43	0.78	1					
PerCP-Cy5.5	0.03	0	0.23	0.04	0.17	0.29	0.35	0.09	0.34	0.09	0.1	0.17	0.01	0.5	0.02	0.21	0.31	0.01	0.39	0.72	0.55	0.25	1				
PerCP	0.03	0.01	0.09	0.05	0.06	0.29	0.13	0.1	0.24	0.1	0.02	0.18	0.01	0.23	0.01	0.08	0.15	0.01	0.38	0.77	0.54	0.27	0.81	1			
Pacific Blue	0.02	0.82	0	0.16	0	0.01	0	0.14	0	0	0	0.06	0.58	0.07	0.36	0.15	0	0.13	0	0	0	0.01	0.01	0.02	1		
FITC	0.55	0.01	0	0.06	0	0.01	0	0.2	0	0.03	0	0.01	0.1	0	0.06	0	0	0.09	0	0.01	0.02	0.06	0	0.01	0.02	1	
APC	0	0	0.05	0.02	0.25	0.27	0.49	0.07	0.87	0.09	0.09	0.14	0	0.23	0.02	0.04	0.24	0	0.78	0.52	0.35	0.16	0.37	0.34	0	0	1

Complexity™ Index: 36.8

Supplemental Figure S1. Antibodies used and Cytek® Full Spectrum Viewer showing calculated similarity indices.

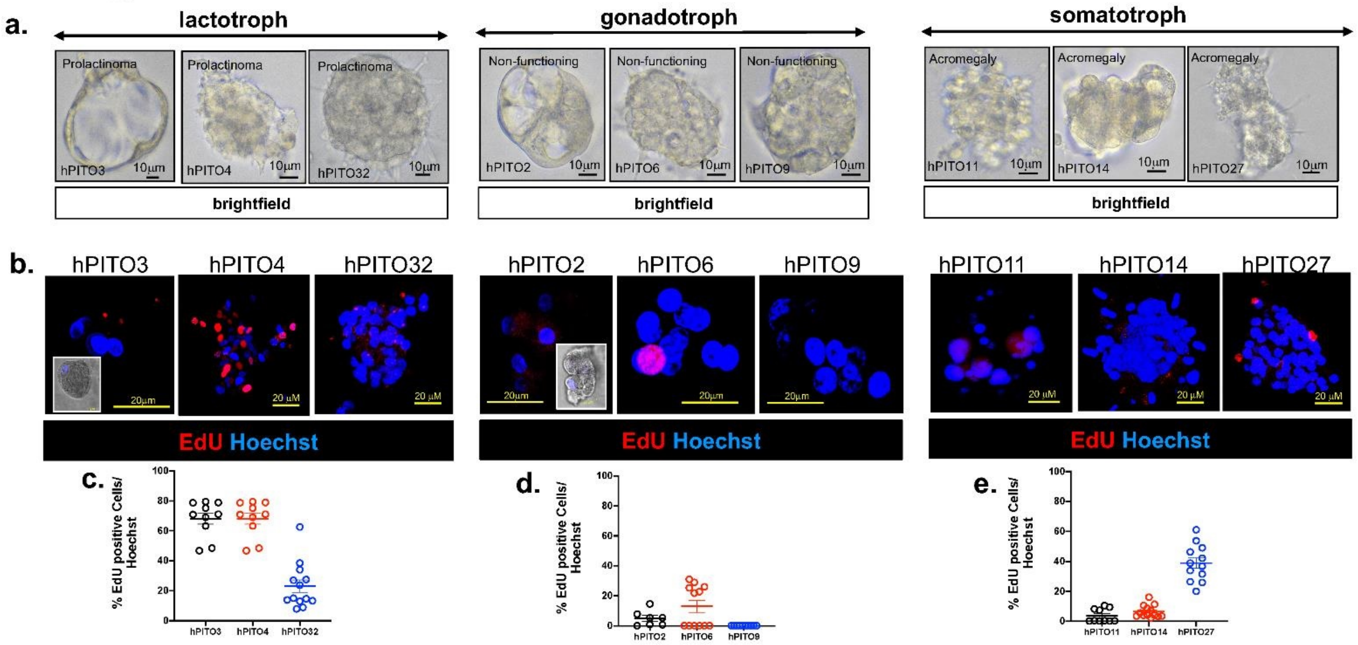

Supplemental Figure S2. Morphology and proliferation of lactotroph, somatotroph and gonadotroph hPITOs. (**a**) Bright-field images, and (**b**) immunofluorescence staining using antibodies specific for EdU (magenta) of organoid cultures gen-erated from patients diagnosed with lactotroph, somatotroph and gonadotroph adenomas. (**c, d, e**) Quantification of % EdU positive cells/ total cell number is shown and compared to the Ki67 score given in the pathology report.

a. iPSC*CDH23*

1. KaryoStat™ analysis of KS-8261 revealed the sample originated from a female individual
2. No chromosomal aberrations were found when comparing against the reference dataset.

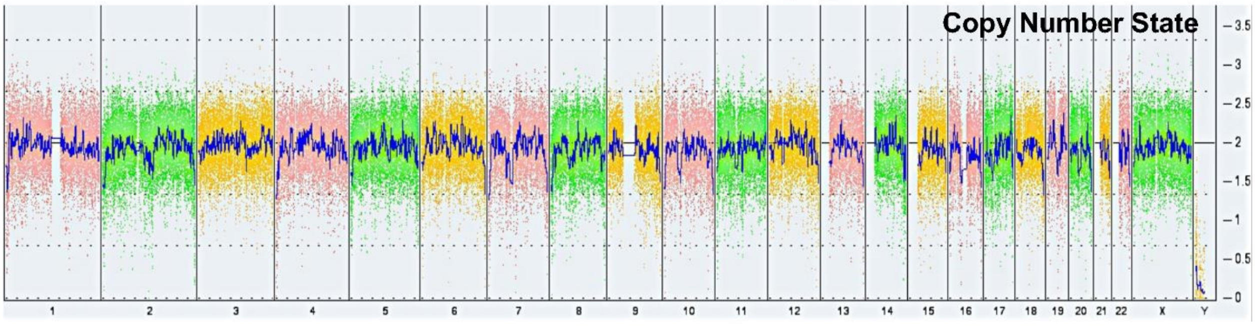

b. iPSC*MEN1*

1. KaryoStat+ analysis of KS-11503 revealed the sample originated from a female individual
2. No chromosomal aberrations were found when comparing against the reference dataset.

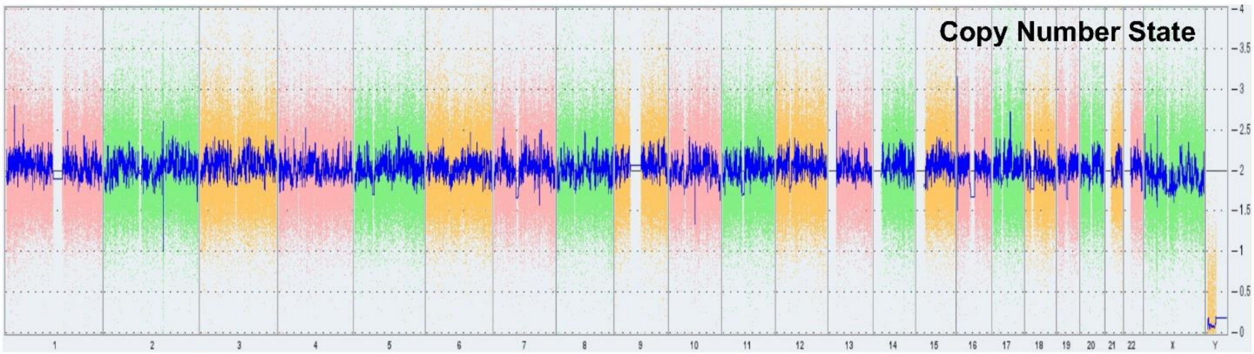

Supplemental Figure S3. KaryoStat™ analysis of (**a**) iPSC^{CDH23} and (**b**) iPSC^{MEN1} lines. No chromosomal aberrations were found in either line when comparing against the reference dataset.

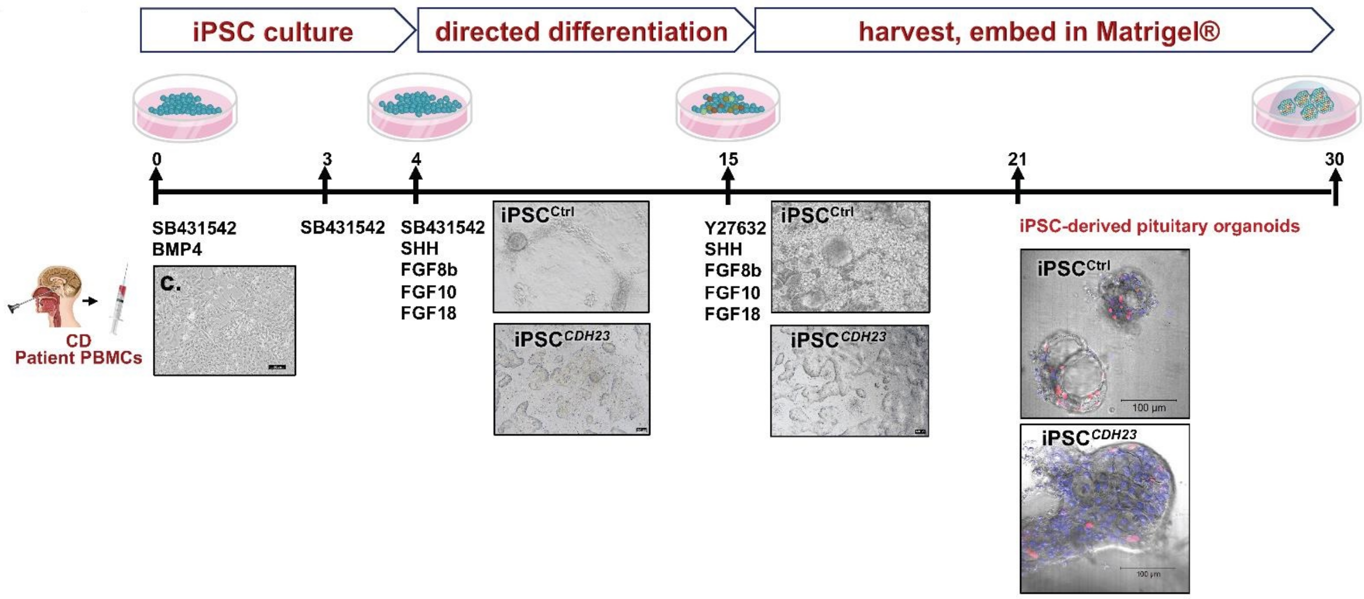

Supplemental Figure S4. Differentiation schedule for the generation of pituitary or ganoids derived from iPSCs. Pituitary organoids were generated based on the outlined schedule using iPSCs generated from PBMCs collected from CD patients, or a healthy individual. Bright field images demonstrating morphological variation is observed between iPSC^{ctrl} and iP-SC^{CDH23} lines and organoids.

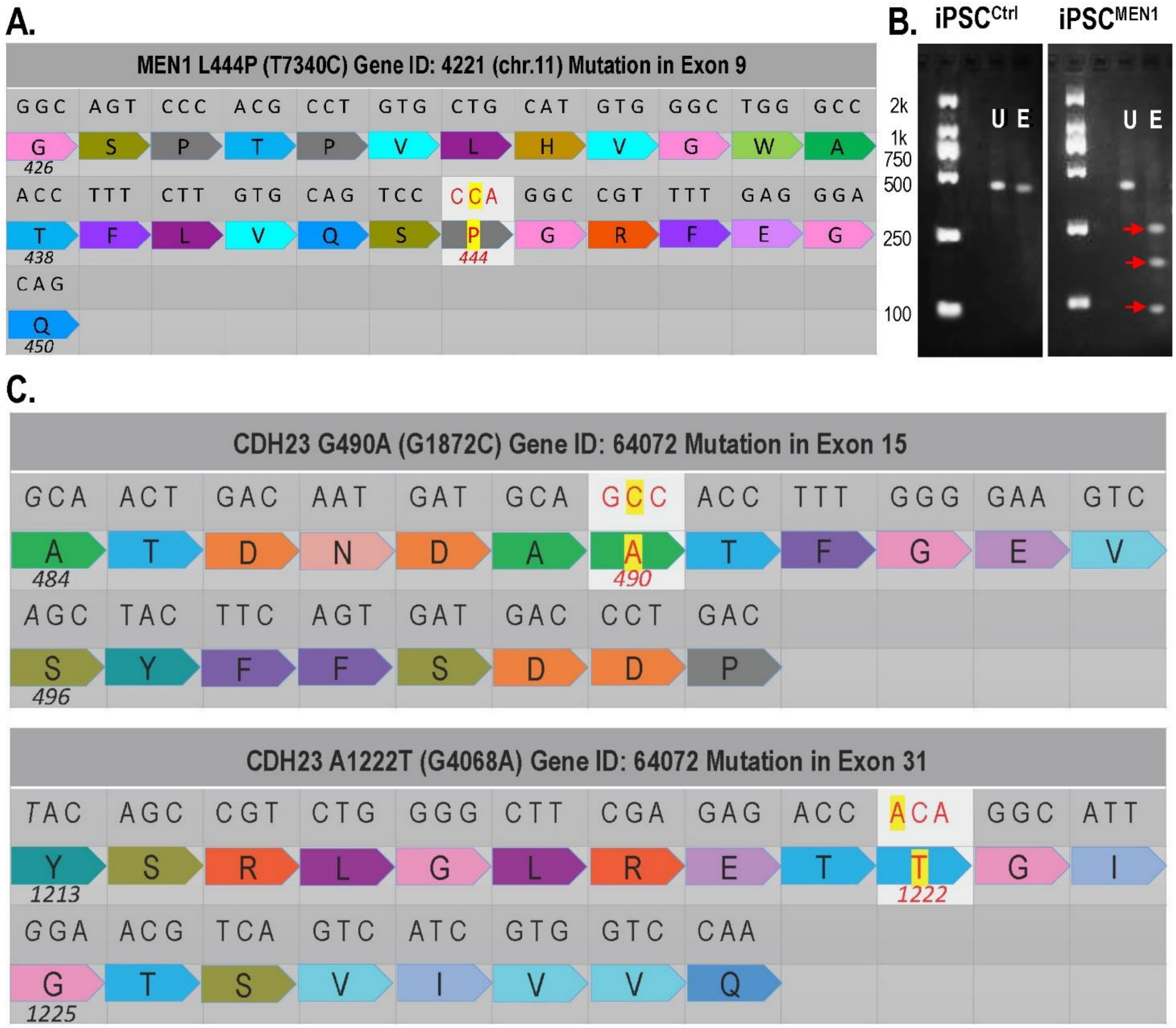

Supplemental Figure S5. Familial germline mutations found in CD patients for the generation of iPSC lines. (A) Amino acid and nucleotide sequence for the germline MEN1 L444P mutation, in exon 9. Also shown are the gene coordinates in the whole MEN1 gene for the subsequence present. (B) Restriction Fragment Length Polymorphism (RFLP) validation to con-firm that iPSC^{MEN1} harbored the correct mutation compared to control. Gel images compared undigested (U) DNA to samples digested with the restriction enzyme EcoRII (E). The MEN1 mutation introduced 2 new EcoRII sites, which is present in the iPSC^{MEN1} gel. iPSC^{Ctrl} displayed no separation of bands in digested versus undigested samples. (C) Amino acid and nucleotide sequence for two germline CDH23 mutations, G490A (exon 15) and A1222T (exon 31) observed in iPSC^{CDH23}. Also shown are the gene coordinates in the entire CDH23 gene for the subsections present.

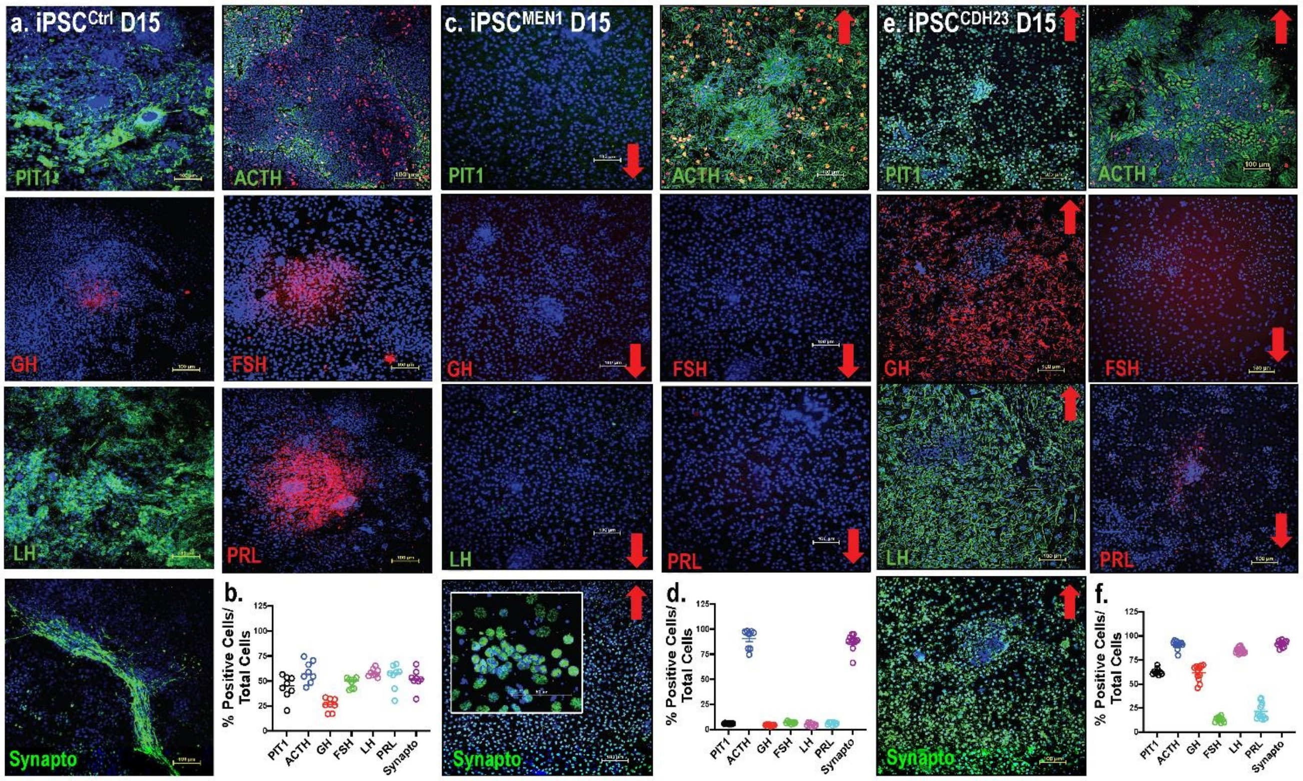

Supplemental Figure S6. Expression pattern of major hormone-producing cell lineages in iPSCs differentiated to pituitary organoids. Expression of PIT-1 (green), ACTH (green), GH (red), FSH (red), LH (green), PRL (red) and synaptophysin (synapto, green) with co-stain Hoechst (nuclei, blue) was measured by immunofluorescence using chamber slides col-lected at day 15 (D15) of the differentiation schedule of control iPSCs ($iPSC^{ctrl}$, **a**, **b**) and iPSCs expressing the MEN1 ($iP\text{-}SC^{MEN1}$ **c**, **d**) and CDH23 ($iPSC^{CDH23}$, **e**, **f**) mutations. Red arrows highlight the increased expression of ACTH and synapto-physin with the concomitant loss of PIT1, GH, FSH, LH and PRL in iPCSs expressing mutated MEN1. Inset in c is a higher magnification of synaptophysin.

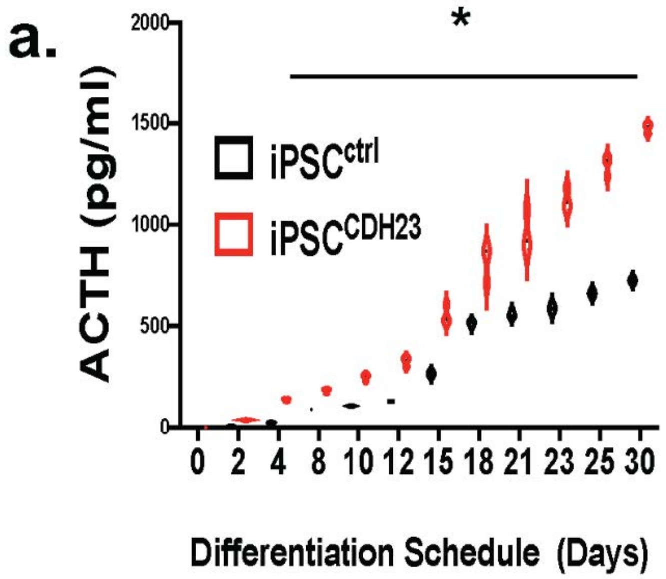

Supplemental Figure S7. Single cell analysis of iPSC^{ctrl} and iPSC^{CDH23} cultures 15 days post-directed differentiation. (**a**) An ELISA was performed using conditioned media collected during the differentiation schedule from iPSC^{ctrl} and iPSC^{CDH23} cultures for the measurement of ACTH secretion (pg/mL). * $P < 0.05$ compared to iPSC^{ctrl} organoid line, n = 3 individual experimental replicates.
