# Supplementary material for: Development of Human Pituitary Neuroendocrine Tumor Organoids to Facilitate Effective Targeted Treatments of Cushing’s Disease
Source: Cells. 2022 Oct 23;11(21):3344. doi: 10.3390/cells11213344 (PMC9659185; doi:10.3390/cells11213344)
Supplement: Supplementary file 1 [file cells-11-03344-s001.zip › cells-1952672-Supplementary Tables.pdf]

Supplemental Table S1. Pituitary Organoid Growth Media.

Component	Stock Conc.	Working Conc.	Catalogue Number
Serum-free defined medium (SFDM)	100%	-	Thermo Fisher Scientific 10744019
B27 (without vitamin A)	1×	2%	Thermo Fisher Scientific 12587010
N2	1×	1%	Thermo Fisher Scientific 17502048
L-Glutamine	200 mM	2 mM	Thermo Fisher Scientific 350-50-061
Penicillin/Streptamycin	100×	1×	Thermo Fisher Scientific 15140122
N-acetyl-L-cysteine	500 mM	1.25 mM	Sigma-Aldrich A7250
Nicotinamide	1 M	10 mM	Sigma-Aldrich N0636
huEGF	500 µg/mL	50 ng/mL	R&D Systems 236-EG200
A83-01 (activin receptor-like kinase (Alk) 4/5/7 inhibitor)	10 mM	0.5 µM	Sigma-Aldrich 2939/10
SB431542 (p38 mitogen-activated protein kinase inhibitor)	10 mM	10 µM	Sigma-Aldrich 1614/10
IGF1	100 µg/mL	100 ng/mL	R&D Systems 291-G1
WNT conditioned media	-	50%	
Rspondin conditioned media	-	10%	
FGF2 (= basic FGF)	20 µg/mL	20 ng/mL	R&D Systems # 233-FB
FGF8	200 µg/ml	200 ng/mL	R&D Systems 423-F8
FGF10	100 µg/mL	100 ng/mL	PeproTech 100-26
FGF18	50 µg/mL	10 ng/mL	R&D Systems 8988-F18
huSHH	200 µg/mL	30 ng/mL	R&D Systems 1845-SH
Noggin	100 µg/mL	100 ng/mL	PeproTech 250-38
Y27632	10 mM	10 mM	Sigma-Aldrich Y0503

Supplemental Table S2. Induced Pluripotent Stem Cell Generated Pituitary Organoid Growth Media.

Component	Catalogue Number	Stock Conc.	Working Conc.
E8 media	UA iPSC core 151169-01	100%	
Penicillin/Streptomycin	Thermo Fisher Scientific 15140122	100×	1×
Y27632	SIGMA Y0503	10 mM	10 uM
E6	Thermo Fisher Scientific A1516401	100%	
BMP4	R&D Systems 314-BP	5 mg/mL	5 ng/mL
SB431542	R&D Systems # 1614/10	10 mM	10 uM
E6	Thermo Fisher Scientific A1516401	100%	
SB431542	R&D Systems # 1614/10	10 mM	10 uM
Human recombinant SHH	R&D Systems 8908-SH	200 ug/mL	30 ng/mL
FGF10	Peprotech 100-26	100 ug/mL	50 ng/mL
FGF8	R&D Systems 423-F8	25 ug/mL	100 ng/mL
FGF18	R&D Systems 8988-F18	10 ng/mL	50 ug/mL

Supplemental Table S3. Clinical Characteristics of Pituitary Adenoma Samples Used for the Gener-ation of Organoids.

Organoid Line	Gender	Age	Neuropathology Report	Clinical Diagnosis	Prior Treatment
**hPITO1**	F	60	Corticotroph subtype, sparsely granulated, T-PIT+, ACTH+, CAM5.2+, synaptophysin+, MIB (Ki67) LI: 1.8%	Cushing's disease	None
**hPITO2**	F	69	Gonadotroph type, SF-1+, alpha subunit+, CAM5.2+, synaptophysin+, MIB (Ki67) LI: 1.2%	Non-functioning	None
**hPITO3**	M	18	Lactotroph type, sparsely granulated (prolacti-noma), PIT-1+, Prolactin+, CAM5.2+, synapto-physin+, MIB (Ki67) LI: <1.0%	Prolactinoma	None
**hPITO4**	M	55	Lactotroph type, sparsely granulated (prolacti-noma), PIT-1+, Prolactin+, CAM5.2+, synapto-physin+, MIB (Ki67) LI: 6.3%	Prolactinoma	None, tumor dis-covered prior to this collection.
**hPITO5****	M	27	Gonadotroph type, SF-1+, alpha subunit+, CAM5.2+, synaptophysin+, MIB (Ki67) LI: <1.0%	Prolactinoma	Cabergoline (2 months prior to this collection)
**hPITO6**	F	87	Gonadotroph type, SF-1+, alpha subunit+, CAM5.2+, synaptophysin+, MIB (Ki67) LI: 2.0%	Non-functioning	None
**hPITO7**	F	53	Corticotroph, Crooke's cell adenoma, T-PIT+, ACTH+, CAM5.2+, CK20+, synaptophysin+, MIB (Ki67) LI: 8.2%	Cushing's disease, ACTH dependent	None
**hPITO8**	F	73	Corticotroph subtype, sparsely granulated, T-PIT+, ACTH+, CAM5.2+, synaptophysin+, MIB (Ki67) LI: 1.0%	Non-functioning, corticotroph type (recurrent)	Prior resection
**hPITO9**	M	71	Gonadotroph type, SF-1+, alpha subunit+, CAM5.2+, synaptophysin+, MIB (Ki67) LI: 1.2%	Non-functioning	None
**hPITO10**	F	34	Corticotroph subtype, sparsely granulated, T-PIT+, ACTH+, CAM5.2+, CK20+, synaptophy-sin+, MIB (Ki67) LI: 1.0%	Cushing's disease	None
**hPITO11**	F	64	Mixed GH/Prolactin cell (biphasic), PIT-1+, Prolactin+, sparsely granulated, GH+ favor in-termediate-type, CAM5.2+, CK20+, SSTR2a dif-fuse strong positivity, E-cadherin+, MIB (Ki67) LI: 1.2%	Acromegaly	None
**hPITO12**	F	55	Corticotroph subtype, sparsely granulated, T-PIT+, ACTH+, alpha subunit scattered+, CAM5.2+, synaptophysin+, MIB (Ki67) LI: <1.0%	Recurrent non-func-tioning	Prior resection for non-functioning pi-tuitary adenoma
**hPITO13**	M	50	Gonadotroph subtype, SF-1+, alpha subunit patchy+, CAM5.2+, synaptophysin+, prolactin+, MIB (Ki67) LI: <1.0%	Non-functioning	None
**hPITO14**	F	62	Pituitary adenoma, PIT-1+, synaptophysin+, CK20+, T-PIT+	Acromegaly	MYCAPSSA (oc-treotide)
**hPITO15**	F	28	Corticotroph, Crooke's cell adenoma, T-PIT+, ACTH+, CAM5.2+, CK20+ highlights Crooke's cell, synaptophysin+, MIB (Ki67) LI: 1.0%	Cushing's disease, ACTH-dependent hypercortisolemia	None

**hPITO16**	M	48	Corticotroph subtype, sparsely granulated, T-PIT+, ACTH+, CAM5.2+, synaptophysin+, alpha subunit+, MIB (Ki67) LI: <1%	Cushing's disease, ACTH-dependent hypercortisolemia	None
**hPITO17**	M	73	Gonadotroph subtype, SF-1+, alpha subunit patchy+, CAM5.2+, synaptophysin+, MIB (Ki67) LI: <1.0%	Non-functioning	None
**hPITO18**	M	44	Gonadotroph subtype, SF-1+, alpha subunit+, CAM5.2+, synaptophysin+, MIB (Ki67) LI: 1.0%	Non-functioning	None
**hPITO19**	F	72	Lactotroph type, sparsely granulated (prolactinoma), PIT-1+, Prolactin+, CAM5.2+, synaptophysin+, alpha subunit+, MIB (Ki67) LI: <1.0%	Prolactinoma	Cabergoline
**hPITO20**	M	38	Acidophil stem cell adenoma, PIT-1+, Prolactin+, GH+, synaptophysin+, CAM5.2 patchy+, CK20+, ACTH+, alpha subunit+, MIB (Ki67) LI: 2.5%	Acromegaly, acidophil stem cell adenoma	None
**hPITO21****	F	53	Lactotroph type, sparsely granulated (prolactinoma), PIT-1+, Prolactin+, synaptophysin+, MIB (Ki67) LI: 1.0%	Prolactinoma	None
**hPITO22**	M	48	Gonadotroph subtype, SF-1+, alpha subunit+, CAM5.2+, synaptophysin+, MIB (Ki67) LI: <1.0%	Non-functioning	None
**hPITO23**	M	66	Gonadotroph subtype, SF-1+, alpha subunit+, PIT-1+, synaptophysin+, thyroid stimulating hormone+, MIB (Ki67) LI: 1.0%	Non-functioning	None
**hPITO24**	M	58	Corticotroph, Crooke's cell adenoma, T-PIT+, ACTH+, CAM5.2+, CK20+, synaptophysin+, MIB (Ki67) LI: 3.5%	Non-functioning	None
**hPITO25**	F	33	Corticotroph subtype, sparsely granulated, T-PIT+, ACTH+, CAM5.2+, synaptophysin+, alpha subunit+, MIB (Ki67) LI: 3.0%	Cushing's disease	None
**hPITO26**	F	46	Gonadotroph subtype, SF-1+, alpha subunit+, CAM5.2+, synaptophysin+, prolactin+, MIB (Ki67) LI: <1.0%	Non-functioning	None
**hPITO27**	F	78	Plurihormonal Pit-1 adenoma with increased proliferation. Prolactin+, GH+, TSH+, alpha subunit+ in some neoplastic cells, ACTH+, Pit-1+, CAM5.2+, synaptophysin+, MIB (Ki67) LI: 11.6%	Acromegaly	History of GH and prolactin secreting PA, prior resection followed by cabergoline and later Mycapssa (octreotide)
**hPITO28**	F	59	Gangliocytoma/mixed gangliocytoma-adenoma. Pit-1+, the larger ganglion-like cells show weak Pit-1 positivity, SF-1+, ACTH+, TPIT+, alpha subunit+, GH+, CAM5.2+, synaptophysin+, MIB (Ki67) LI: <1%	Cushing's, ACTH-dependent hypercortisolemia	None
**hPITO29****	M	27	Lactotroph subtype, sparsely granulated (prolactinoma). PIT-1+, Prolactin+, CAM5.2+, synaptophysin+, alpha subunit+, MIB (Ki67) LI: <1.0%	Prolactinoma	Cabergoline

**hPITO30****	F	53	Corticotroph subtype, sparsely granulated, T-PIT+, ACTH+, CAM5.2+, synaptophysin+, prolactin+, PIT-1, MIB (Ki67) LI: 1%	Cushing's disease, recurrent	Mifepristone
**hPITO31**	F	67	Corticotroph subtype, sparsely granulated, T-PIT+, ACTH+, CAM5.2+, synaptophysin+, CK20+, MIB (Ki67) LI: <1%	Non-functioning	None
**hPITO32**	F	39	Actotroph type, sparsely granulated (prolactinoma), T-PIT/SF-1+, PIT-1+, Prolactin+, CAM5.2+, synaptophysin+, alpha subunit+, MIB (Ki67) LI: 1–2%	Prolactinoma vs Rathke's cleft cyst (former favored)	Cabergoline
**hPITO33**	F	57	Crooke cell adenoma. T-PIT+, ACTH+, CAM5.2+, CK20+, synaptophysin+, MIB (Ki67) LI: <1%	Cushing's disease	None
**hPITO34**	M	67	Corticotroph subtype, sparsely granulated. T-PIT+, ACTH+, CAM5.2+, CK20+, synaptophysin+, alpha subunit+, MIB (Ki67) LI: <1%	Cushing's disease	None
**hPITO35**	F	54	Corticotroph subtype, sparsely granulated, T-PIT+, ACTH+, CAM5.2+, CK20+, synaptophysin+, alpha subunit+, MIB (Ki67) LI: <1%	Cushing's disease	None
**hPITO36**	F	45	Corticotroph subtype, sparsely granulated, T-PIT+, ACTH+, CAM5.2+, CK20+, synaptophysin+, alpha subunit+, MIB (Ki67) LI: 1.3%	Cushing's disease	Prior resection for nonfunctioning pituitary adenoma
**hPITO37**	F	53	Crooke cell adenoma. T-PIT+, ACTH+, CAM5.2+, CK20+, synaptophysin+, MIB (Ki67) LI: <1%	Cushing's disease	None
**hPITO38**	F	43	Corticotroph subtype. T-PIT+, ACTH+, CAM5.2+, CK20+, synaptophysin+, MIB (Ki67) LI: <1%	Cushing's disease	None
**hPITO39**	M	62	Corticotroph subtype, sparsely granulated, T-PIT+, ACTH+, CAM5.2+, CK20+, synaptophysin+, MIB (Ki67) LI: <1%	Cushing's disease	None
**hPITO40**	F	28	Corticotroph subtype, Crooke cell adenoma, T-PIT+, ACTH+, CAM5.2+, CK20+, synaptophysin+, alpha subunit+, MIB (Ki67) LI: 2.5%	Cushing's disease	None

**: Organoid culture not established or could not be maintained/expanded.

Supplemental Table S4. Average Correlation of Replicates Reported in Figure 3.

Organoids	Average Correlation of Replicates
hPITO16	0.98
hPITO35	0.99
hPITO34	1
hPITO33	0.99
hPITO28	0.92
hPITO7	0.81
hPITO10	0.22
hPITO25	0.97

Supplemental Table S5. Pituitary Cell Lineage or Stem Cell Markers used in the scRNA-seq Analy-sis.

Pathway	Genes
STEM CELL	SOX2, PROP2, MIK67, S100B, SCA1, KIT, CD34, PROM1, POU5F1, NANOG, NES, BMI1, SOX4, SOX9, NOTCH2, HES1, PROP1, CDH1, CDH2, CDH3, NR5A1
NOTCH SIGNALLING	NOTCH, NOTCH1, NOTCH2, HES1, DTX4
WNT SIGNALLING	WNT, FZD8, FZD2, WNT5A, SFRP2, TCF7L1, FZD7,
HEDGEHOG	SHH, PTCH1, SMO, GLI1, GLI2, GLI3, GAS1, PRKX,
TIGHT JUNCTION	CLDN4, CLDN5, TJP2, CLDN16, CDH1 (Epithelial - related to stem cells)
ECM_RECEPTOR	VIM, CDH2, LAMB1, LAMB2, FN1, COL5A1 (Mesenchymal - related to stem cells)
TGF_BETA	ID1, ID2, ID3, ID4, SMAD3, BMP2,
EPITHELIAL	EPCAM, CDH1, KRT8, CLDN4, GRHL2,
MESENCHYMAL	CDH2, VIM, COL1A1, COL1A2, SNAI1,
PIT1	POU1F1, GH2, PRL, TSHB, GLVR1, Glvr-1, PIT1, PiT-1
CORTICOTROPH	TBX19, POMC, PAX7, NEUROD1, PITX1, AGSU, LIF, AR, FST, NR4A1, NR4A2,
SOMATOTROPH	PIT1, PRL, GHB, GHRH, GHRHR, POU1F1, SP1, NEUROD4, FOXO1, NKX2, NKX2-2, SOX11, ZBTB20, MATH3,
LACTOTROPH	NEUROD4, SOX11, ZBTB20, POU1F1, PRL, MAPK, PITX1, PITX2,
THYROTROPH	NEUROD4, SOX11, ZBTB20, POU1F1, GATA2, TSHB, LHX3,
GONADOTROPH	GATA2, NEUROD1, NR5A1, POU1F1, SF1, CGB3, CGB5, CYP11A1, WNT4, MC2R,, FOLR1, SCGB2A1, LHB, FSHB, EGR1,
HYPOTHALAMUS	BRN2, OTP, SIM1, SIM2, ARNT1, HMX2, HMX3, GSH1, NHLH2, EBF2,
SST SIGNALLING	SST, SSTR1, SSTR2, SSTR3, SSTR4, SSTR5 ;
PROLIFERATION	CDK1, CDK2, CDK4, CDK6, MKI67, CCNB1, CCNB2, E2F1, E2F7, RBP1, RBP4, RBP7
