# Supplementary material for: Development of Human Pituitary Neuroendocrine Tumor Organoids to Facilitate Effective Targeted Treatments of Cushing’s Disease
Source: Cells. 2022 Oct 23;11(21):3344. doi: 10.3390/cells11213344 (PMC9659185; doi:10.3390/cells11213344)
Supplement: Supplementary file 1 [file cells-11-03344-s001.zip › cells-1952672-Supplementary Videos/cells-1952672-Supplemental Video Legends.pdf]

Supplemental Video 1: 3D rendering of a confocal image captured from CD PitNET tissue derived organoid culture. Movie of z-stack captured through the hPITO37 immunofluorescently stained for CAM5.2 (red) and ACTH (green). Hoechst was used for nuclear staining (blue).

Supplemental Video 2: Time-lapse video of iPSC PitNET derived organoid culture (area 1). Video of live time-lapse brightfield images, captured by a Nikon ECLIPSE Ti2 microscope, of organoid culture scanned automatically every 30 minutes for 72 hours.
